# Novel RNA- and FMRP-binding protein TRF2-S regulates axonal mRNA transport and presynaptic plasticity

**DOI:** 10.1038/ncomms9888

**Published:** 2015-11-20

**Authors:** Peisu Zhang, Kotb Abdelmohsen, Yong Liu, Kumiko Tominaga-Yamanaka, Je-Hyun Yoon, Grammatikakis Ioannis, Jennifer L. Martindale, Yongqing Zhang, Kevin G. Becker, In Hong Yang, Myriam Gorospe, Mark P. Mattson

**Affiliations:** 1 Laboratory of Neurosciences, National Institute on Aging Intramural Research Program, National Institutes of Health, 251 Bayview Boulevard, Baltimore, Maryland 21224, USA; 2 Laboratory of Genetics, National Institute on Aging Intramural Research Program, National Institutes of Health, 251 Bayview Boulevard, Baltimore, Maryland 21224, USA; 3 Department of Biomedical Engineering, National University of Singapore, Singapore 117465, Singapore; 4 SINAPSE, National University of Singapore, Singapore 117465, Singapore; 5 Department of Neuroscience, Johns Hopkins University School of Medicine, Baltimore, Maryland 21205, USA

## Abstract

Despite considerable evidence that RNA-binding proteins (RBPs) regulate mRNA transport and local translation in dendrites, roles for axonal RBPs are poorly understood. Here we demonstrate that a non-telomeric isoform of telomere repeat-binding factor 2 (TRF2-S) is a novel RBP that regulates axonal plasticity. TRF2-S interacts directly with target mRNAs to facilitate their axonal delivery. The process is antagonized by fragile X mental retardation protein (FMRP). Distinct from the current RNA-binding model of FMRP, we show that FMRP occupies the GAR domain of TRF2-S protein to block the assembly of TRF2-S–mRNA complexes. Overexpressing TRF2-S and silencing FMRP promotes mRNA entry to axons and enhances axonal outgrowth and neurotransmitter release from presynaptic terminals. Our findings suggest a pivotal role for TRF2-S in an axonal mRNA localization pathway that enhances axon outgrowth and neurotransmitter release.

## Abstract (web summary)

The molecular mechanisms regulating axonal mRNA transport are only partially understood. Here, Zhang *et al*. show a nontelomeric TRF2 splice variant interacts with FMRP to regulate the transport of several axonal mRNAs involved in axonal elongation and neurotransmitter release.

## Graphical abstract

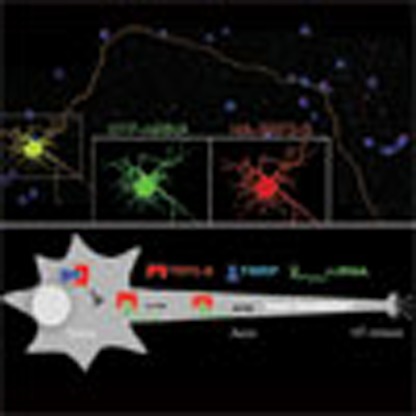

Since the early discovery of polyribosomes in the base of dendritic spines^1^, the mechanisms underlying the local control of protein synthesis became an area of focus in modern neurobiology^2^. The spatially restricted regulation of protein translation is believed to play fundamental roles in synaptic plasticity and cognitive function^2^,^3^,^4^. In addition to protein synthesis in the cell body of neurons, certain proteins are synthesized locally using messenger RNAs that are selectively transported into dendrites and axons^2^,^3^. The mRNAs located in neurites can then be translated repeatedly to produce high concentrations of proteins in response to synaptic activation. Recent findings suggest that axons may deploy local translation of mRNAs to regulate axon outgrowth and regeneration, and synapse formation and remodelling^5^,^6^,^7^,^8^. Both developing^8^ and mature^9^,^10^,^11^ axons contain specialized mRNA repertoires and associated molecular machineries^5^,^12^ that have been proposed to enable local translation of mRNAs in growth cones and presynaptic terminals^6^,^13^,^14^. For example, Taylor *et al*.^15^ reported that locally translated β-catenin accumulates at presynaptic terminals of cultured hippocampal neurons where it may regulate neurotransmitter release. However, roles for RNA-binding proteins (RBPs) in regulating axonal plasticity in the developing and adult nervous system are largely unknown.

Intracellular mRNA trafficking is facilitated by the formation of ribonucleoprotein (RNP) complexes via binding of RBPs to specific mRNA motifs^3^,^16^,^17^. Among neuronal RBPs, fragile X mental retardation protein (FMRP) has emerged as a pivotal regulator of local protein synthesis, especially in dendrites. Fragile X syndrome, the most common inherited cognitive deficit disorder, is caused by loss of function of FMRP. FMRP usually binds to a subgroup of dendritic mRNAs^18^ and acts as a translational break by stalling ribosomes on the mRNAs^19^. Neurons lacking FMRP typically exhibit excessive protein synthesis and aberrant growth of dendritic spines^20^,^21^. Recent findings suggest that FMRP is also located in axons of immature vertebrate neurons, where it may influence growth and neurotransmitter release^22^ by inhibiting translation^23^,^24^. However, the function of FMRP in mature vertebrate axons has yet to be elucidated.

In proliferating cells, full-length telomere repeat-binding factor 2 (TRF2) binds and protects telomeres with its carboxy-terminal Myb domain^25^,^26^. Interestingly, TRF2 also binds to a non-coding telomeric RNA (TERRA) via an amino-terminal glycine–arginine-rich (GAR) domain in TRF2 (ref. 27). The GAR domain, also known as RGG box, is a common RNA-binding motif present in several RBPs such as FMRP^28^ and nucleolin^29^. Recently, we discovered a non-telomeric splice variant of TRF2 (TRF2-S) expressed in postmitotic neurons^30^. TRF2-S retains the N-terminal GAR domain but lacks the C-terminal Myb DNA-binding domain of TRF2, suggesting a plausible switch of binding preference from DNA to RNA.

Here we report that TRF2-S is a neuron-specific RBP that directs the entry of selective mRNAs into mature axons. We demonstrate opposing actions of TRF2-S and FMRP in regulating the anterograde transport of axonal mRNAs and axonal plasticity. The opposing actions of FMRP and TRF2-S on the axonal mRNAs results from an interaction of the two RBPs, rather than from their competing for common targeting motifs in mRNAs. The TRF2-S GAR domain serves as the binding site not only for the axonal mRNAs, but also for FMRP; binding of FMRP to TRF2-S inhibits the assembly of TRF2-S–mRNA complexes, which in turn retards the axonal transport of mRNAs. Finally, our data indicate that TRF2-S promotes, whereas FMRP inhibits axon outgrowth and neurotransmitter release from presynaptic terminals.

## Results

### TRF2-S associates with mRNAs of cortical neurons

**Figure 1:**
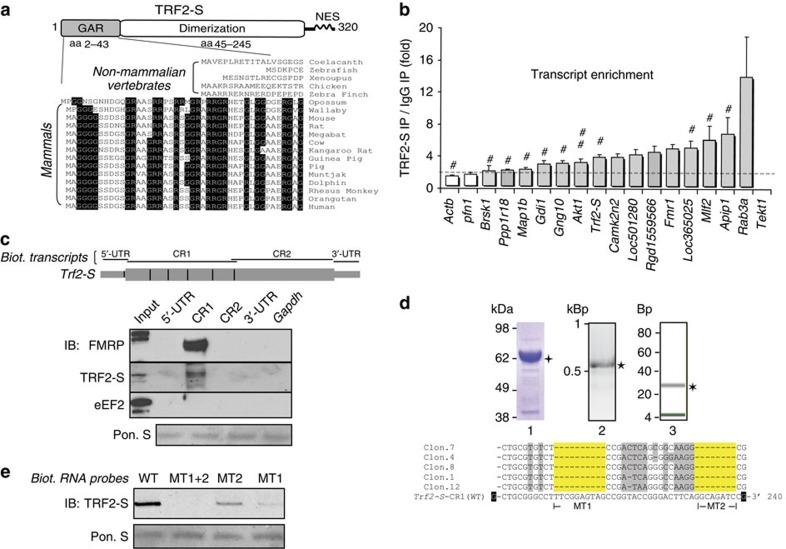
Neuronal isoform of TRF2-S exhibits RNA-binding activity. (**a**) Top, schematic representation shows that TRF2-S contains a GAR RNA-binding domain. NES, nuclear export signal. Bottom, the alignment of the GAR domains from vertebrate TRF2-S shows that glycine–arginine consensus sequences (highlighted in black) are highly conserved across mammals but not in non-mammalian species. (**b**) Validation of TRF2-S target mRNAs by quantitative RT–PCR. Among 18 transcripts, 15 (grey bars) were enriched more than 2-fold by TRF2-S IP over IgG IP controls. *n*=4. #known axonal mRNA; ##self mRNA. (**c**) Biotin pulldown and immunoblotting showing that TRF2-S bound to the coding region (CR1) of *Trf2-S* mRNA. Biotinylated mRNA fragments of *Trf2-S* were transcribed *in vitro* and incubated with rat brain lysates. Biotinylated *Gapdh* RNA and eukaryotic elongation factor 2 (eEF2) are controls for biotin pulldown and immunoblot analysis, respectively. Equal loading was assessed by Ponceau S staining. The mRNA fragments and guanine (G)-rich sequences were mapped as horizontal and vertical lines in the upper diagram, respectively. (**d**) *In vitr*o mapping of *Trf2-S* binding site for TRF2-S. Upon ultraviolet cross-linking, the covalent bound RNP complex of recombinant GST–TRF2-S protein (line 1) and *Trf2-S*-CR1(★ line 2) transcript was collected after RNase T1 treatment and GST purification. The purified small RNAs (∼30 nt, line 3) were then used for cDNA library preparation, cloning and sequencing. Bottom, the representative sequenced reads of 11 clones were distinct from the parental *Trf2-S* WT. Yellow highlights, putative *Trf2-S* mRNA-binding sites for TRF2-S; black highlights, 3′-end of guanine residues expected from RNase T1 digestion; grey highlights, base substitutions and deletions expected from ultraviolet irradiation. (**e**) Biotinylated WT and mutant RNA oligos (MT1, MT2 and MT1+2 of TRF2-S-binding sites) were incubated individually with the purified recombinant GST–TRF2-S for biotin pulldown and immunoblot analysis.

In the evolutionary transition from non-mammalian vertebrates to mammals, TRF2 and TRF2-S proteins acquired an RNA-binding motif^31^ with a highly conserved GAR domain (Fig. 1a). Distinct from its telomere-binding counterpart TRF2, TRF2-S lacks a DNA-binding domain, suggesting that TRF2-S may shift its binding preference from DNA to RNA.

To identify global *in vivo* interactions of TRF2-S with mRNAs in neurons, we initiated the study with a procedure known as an RNP immunoprecipitation (RIP) assay^29^. We used a previously validated TRF2-S antibody^30^ to co-immunoprecipitate RNAs from the extracts of cortical neurons (9 days in culture). The mRNA species enriched in the TRF2-S–RIP precipitates were extracted and then identified by Illumina microarray analysis. A parallel control IP was performed using IgG. Analysis of microarray data sets yielded a list of 140 transcripts that were highly enriched in the TRF2-S RIP with *z*-ratios >1.8 (Supplementary Table 1). To elucidate the potential functions of TRF2-S, the microarray data set (NCBI GEO accession numbers: GSE72887 and GSM1874108–GSM187414) was submitted to DAVID analysis (http://david.abcc.ncifcrf.gov/) and the bound transcripts were categorized using a functional annotation clustering feature to identify gene families that were highly enriched (*P*<0.001). The top-ranked Gene Ontology terms in TRF2-S-bound transcripts were in categories associated with various aspects of neuronal structure and function, including mitochondrial membrane, axonogenesis and microtubule cytoskeleton dynamics (Supplementary Fig. 1a). We also tested a ultraviolet cross-linking-based IP (photoactivatable-ribonucleoside-enhanced cross-linking and IP) assay^32^; however, this was unsuccessful.

To validate the microarray results, we performed reverse transcription (RT) followed by real-time, quantitative PCR (qPCR) to amplify TRF2-S-bound transcripts encoding proteins within several categories of neuronal functions including the following: axonal transport and regulation of neurotransmitter release (*Rab3a*, *Aplp1* and *Gdi1* mRNAs); cytoskeletal dynamics (*Tekt1*, *Loc365025/alpha tubulin*, *Loc501280/Myosin reg. LC*, *Map1b*, *Pfn1* and *Actb*); protein translation (*Rgd1559566*/*60S/L9)*; calcium-mediated signal transduction (*Camk2n2*, *Akt1*, *Ppp1r18* and *Brsk1*); nitric oxide synthase and G protein signalling (*Mlf2* and *Gng10*); and two known FMRP target transcripts (*Fmr1* and *Map1b*)^33^. In comparison with IgG IP, TRF2-S IP showed more than 2-fold enrichment in 15 out of 18 TRF2-S target mRNAs (Fig. 1b).

As ‘axonogenesis' stood out as an enriched functional category, we evaluated axonal attributes in TRF2-S-bound mRNAs. We compared our data sets with an axonal mRNA library where the mRNA populations were extracted from axons grown in compartmentalized microfluidic chambers. Notably, 34 of the 140 TRF2-S target transcripts were included in the axonal transcriptomes^11^ (Supplementary Table 2 and Supplementary Fig. 1b) and several axonal mRNAs, including *Rab3a*, *Aplp1*, *Mlf2*, *Camk2n2*, *Akt1*, *Gng10*, *Gdi1* and *Map1b* mRNAs were indeed enriched in TRF2-S–RNP complexes (Fig. 1b).

### Mapping of TRF2-S mRNA-binding footprints in *Trf2-S*

The fact that RBPs often recognize their own mRNAs^34^ led us to determine how TRF2-S bound to *Trf2-S*. We employed an *in vitro* protein–mRNA binding assay (biotin pulldown assay)^29^,^35^ to identify *Trf2-S–*RNP complexes using the cytoplasmic lysate of rat brains homogenized in a high-salt Co-IP buffer with 450 mM KCl. A set of biotinylated RNA fragments were synthesized to span the entire *Trf2-S* transcript (Fig. 1c). As it has been established that RBPs such as TRF2 (ref. 27) and FMRP^28^,^35^ harbour GAR or RGG domains that recognize G-rich RNA structures known as G-quartets, we performed the *in silico* analysis using QGRS Mapper (http://bioinformatics.ramapo.edu/QGRS/) to search for putative G-quartets and their location within the *Trf2-S* transcript (Fig. 1c). We found that FMRP and TRF2-S in brain lysates were pulled down together by the same G-rich coding region of *Trf2-S* (region CR1).

To elucidate the precise TRF2-S mRNA-binding footprint in *Trf2-S*, we performed an *in vitro* ultraviolet cross-linking and purification assay. On covalent cross-linking of recombinant glutathione *S*-transferase (GST)–TRF2-S protein with the *Trf2-S*-CR1 transcript, RNase T1-resistant small RNAs were subjected to GST purification, complementary DNA library preparation, cloning and sequencing. We identified 11 out of 19 clones that contained the appropriate sequenced reads that corresponded to ultraviolet cross-linking and RNase T1 treatments (Fig. 1d). Interestingly, the data revealed two distinct binding sites in *Trf2-S* mRNA that were potentially correlated with TRF2-S occupancy. We then synthesized a set of wild-type (WT) and mutant RNA probes. to validate their binding to GST–TRF2-S. The WT probe covers 47 nt of *Trf2-S* aligned with the sequenced reads. The mutant probes were engineered at the TRF2-S-binding sites with mismatched bases (A replaced with T, G replaced with C and *vice versa*). Using the biotin pulldown assay with these RNA probes, we found that both binding sites were indispensable for TRF2-S recognition, but the MT1 region displayed a higher binding capability compared with MT2 (Fig. 1e).

### The TRF2-S GAR domain binds either to mRNAs or to FMRP

**Figure 2:**
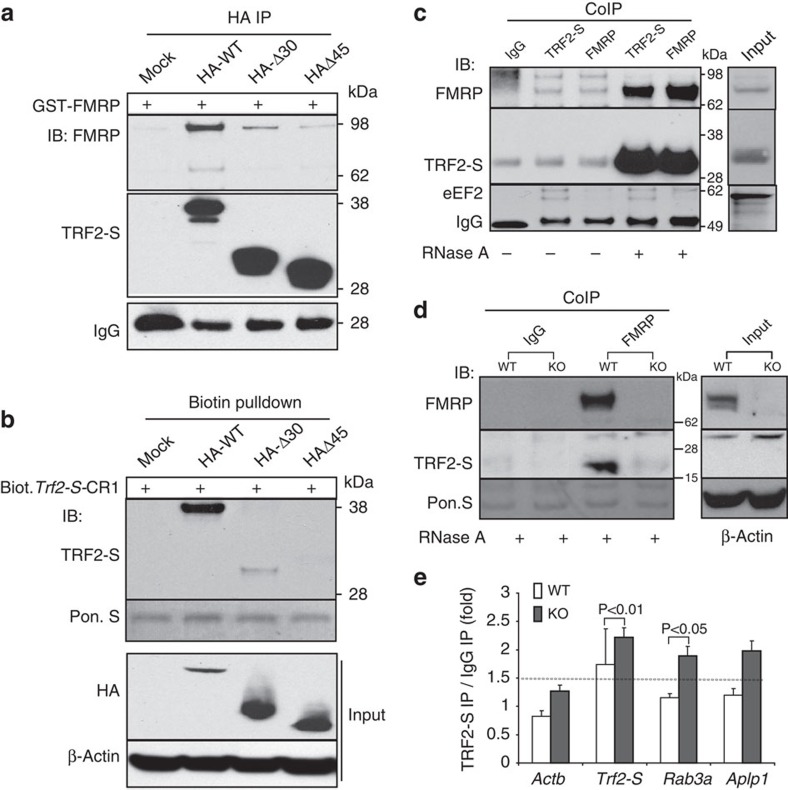
The TRF2-S GAR domain recruits either FMRP or *Trf2-S* mRNAs. (**a**) *In vitro* Co-IP showing that TRF2-S GAR domain is required for recruiting FMRP. Lysate from HEK293 cells expressing HA–TRF2-S WT or mutants with deletion of amino acids 1–30 (HA-▵30) or 1–45 (HA-▵45) of the GAR domain were incubated with purified recombinant GST–FMRP before HA–Co-IP. The abilities of HA–TRF2-S variants to recruit FMRP were analysed by immunoblotting. Equal loading was assessed by IgG. (**b**) TRF2-S GAR domain is indispensable for binding *Trf2-S* mRNA. Upon HA immunopurification, the binding of HA–TRF2-S variants to *Trf2-S*-CR1 mRNA were assessed by biotin pulldown analysis. Equal loadings were assessed using Ponceau S and β-actin. (**c**) Co-IP of endogenous TRF2-S and FMRP using brain cytoplasmic extract in the presence or absence of RNase A. The immunoblots showing that the binding of TRF2-S to FMRP, but not to eukaryotic elongation factor 2 (eEF2), was remarkably enhanced by RNA elimination. Equal loading was assessed by IgG level. (**d**) FMRP Co-IP using WT and *Fmr1* KO mice brains. Equal loading was assessed by β-actin western blotting and Ponceau S staining for inputs and precipitates, respectively. (**e**) *In vivo* TRF2-S–RIP using WT and *Fmr1* KO cerebella. The enrichments of TRF2-S target mRNAs were assessed by RT–qPCR. The values are the fold change normalized to *18S* rRNA by comparisons of TRF2-S IP with IgG IP. Dotted line, 1.5-fold change; *n*=4 per genotype. All values are mean±s.d. *P*-values based on Student's *t*-test.

As biotinylated *Trf2-S*-CR1 pulled down not only TRF2-S but also FMRP from brain lysates, it is possible that FMRP can interact with either TRF2-S target mRNA or TRF2-S protein, or both, thereby influencing the formation of TRF2-S–RNP complexes. To test these possibilities, we first examined in parallel whether the TRF2-S GAR domain is responsible for recruiting FMRP or TRF2-S-bound mRNA. We co-immunoprecipitated a purified recombinant GST–FMRP protein with haemagglutinin (HA)–TRF2-S WT and two mutants engineered for partial (HA-▵30) or complete deletion (HA-▵45) of the GAR domain. Compared with TRF2-S WT, the binding of TRF2-S mutants to FMRP was progressively reduced as the GAR deletions were extended (Fig. 2a). Similarly, the results from biotin pulldown showed that the binding of *Trf2-S*-CR1 was greatly diminished with the HA-▵30 mutant and undetectable with the HA-▵45 mutant (Fig. 2b). These data suggest that the binding sites in TRF2-S for recruiting FMRP and *Trf2-S* mRNA overlap.

Next, we determined whether the presence of RNA could interrupt the interaction of TRF2-S and FMRP. A previous study indicated that either high-salt buffers or RNase treatment are necessary for the disassociation of FMRP from larger RNA–protein complexes^36^. We found that TRF2-S bound to FMRP sufficiently under a high-salt (450 mM KCl) IP condition (Supplementary Fig. 2a). For assessing RNA effects, we therefore employed 150 mM KCl low-salt buffer to homogenize the brain tissue, then treated the lysate either with or without RNase A before TRF2-S and FMRP Co-IP. Upon elimination of RNA, TRF2-S and FMRP were readily detected in their reciprocal immunoprecipitates (Fig. 2c). Interestingly, the immunoprecipitates of TRF2-S and FMRP were reduced considerably when RNase treatment was omitted from the protocol, suggesting that the presence of RNA inhibited Co-IP of TRF2-S and FMRP. However, such conditions apparently had no effect on Co-IP of TRF2-S with an RBP, eukaryotic elongation factor 2. The results indicated that the bindings of TRF2-S to FMRP, and to RNA, are biochemically distinct.

To confirm the findings, we took advantage of the well-characterized *Fmr1* knockout (KO) mouse^19^,^22^,^35^ to determine whether FMRP was dispensable for the binding of TRF2-S to its mRNA targets *in vivo*. Brain tissue samples from *Fmr1* KO and WT mice were homogenized and then treated with RNase A for RNA removal before FMRP Co-IP. We first found that TRF2-S expression was enhanced in Fmr1 KO brain in comparison with the WT counterpart (Supplementary Fig. 2b). We then observed that an FMRP antibody pulled down TRF2-S from WT brain lysates but not from Fmr1 KO brain lysates (Fig. 2d). On the other hand, a TRF2-S antibody was able to enhance the RIP precipitation of TRF2-S mRNA targets, *Trf2-S*, *Aplp1* and *Rab3a*, from Fmr1 KO brain tissue but not from WT mouse brain tissue (Fig. 2e). The data demonstrated that TRF2-S by itself was able to bind to these mRNAs in the absence of FMRP and also suggested a specific binding of FMRP to TRF2-S that might inhibit the ability of TRF2-S to bind to RNAs. Interestingly, we also observed that TRF2-S RIP from WT mice exhibits a weaker signal than from primary cultured neurons, which is consistent with expression of TRF2-S in the neurons, but not in the glia^30^, probably resulting in a dilution effect in samples from the brain compared with pure neuronal cultures.

### TRF2-S binds directly to axonal mRNAs

**Figure 3:**
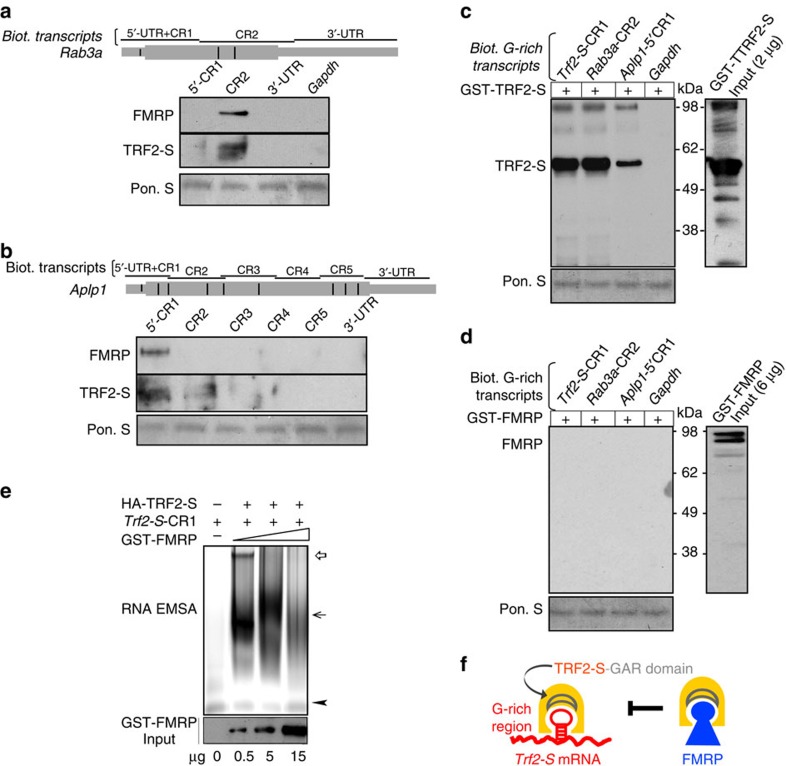
TRF2-S but not FMRP interacts directly with axonal mRNAs. (**a**,**b**) Biotin pulldown and immunoblotting showing that the axonal mRNA fragments derived from the coding regions of *Rab3a*-CR2 and *Aplp1*-5′CR1 pulled down FMRP and TRF2-S proteins from rat brain lysate. (**c**,**d**) Western blot analysis to assess whether TRF2-S or FMRP bound directly to the G-rich RNA fragments. Three biotinylated G-rich mRNA fragments (*Trf2-S*-CR1, *Rab3a*-CR2 and *Aplp1*-5′CR1) pulled down purified recombinant GST–TRF2-S (**c**), but not GST–FMRP (**d**). (**e**) RNA mobility shift assay (EMSA) showing that binding of FMRP to TRF2-S inhibited the formation of TRF2-S–mRNA complex. Purified HA–TRF2-S (4.6 μg) was incubated with *Trf2*-S-CR1 RNA probe (2 μg) and indicated amounts of GST–FMRP protein for RNA EMSA. The upper open and closed arrows point to bound RNAs and the lower arrowhead points to unbound RNA. (**f**) Model: FMRP inhibits the binding of TRF2-S to its target mRNAs.

To elucidate the biological function of TRF2-S RNA-binding activity, we determined whether TRF2-S binds the axonal mRNAs *Rab3a* and *Aplp1*, which encode proteins that regulate presynaptic vesicle trafficking and neurotransmitter release^37^,^38^. Consistent with the results of the *Trf2-S* biotin pulldown, the biotinylated mRNA fragments derived from *Rab3a*-CR2 and *Aplp1*-5′CR1 were also able to precipitate both FMRP and TRF2-S proteins from rat brain lysate (Fig. 3a,b).

To determine whether TRF2-S and/or FMRP bind directly to TRF2-S RNA targets, we synthesized purified recombinant GST–TRF2-S and GST–FMRP to perform parallel biotin pulldown assays using three G-rich RNA fragments *Trf2-S*-CR1, *Aplp1*-5′CR1 and *Rab3a*-CR2, and *Gapdh* as a negative control. We found that these G-rich RNA fragments efficiently pulled down GST–TRF2-S but not GST–FMRP (Fig. 3c,d). To confirm the finding, we replaced GST–FMRP with GST–FMRP C terminus (ct) that contains an RNA-binding RGG box (Supplementary Fig. 3a). The latter also failed to bind any of the three RNA fragments. As an additional control, we indeed observed that GST–FMRP was capable of binding to two *App* mRNA fragments that harbour known FMRP target sites (Supplementary Fig. 3b)^39^,^40^.

To further interrogate the interactions of TRF2-S and FMRP with RNA, we determined whether FMRP competes with *Trf2-S* mRNA for binding to TRF2-S. We incubated HA–TRF2-S-purified protein and *Trf2*-S CR1 RNA with varied titrations of recombinant GST–FMRP for an RNA mobility-shift assay. We observed that elevating GST–FMRP levels inhibited the formation of TRF2-S–RNP complexes (Fig. 3e). We also found that HA-TRF2-S but not GST–FMRP shifted TRF2-S target RNAs, whereas premixing GST–FMRP protein with HA–TRF2-S resulted in a substantial reduction of a slower-migrating species of the TRF2-S–RNP complex (Supplementary Fig. 2c). The latter data are consistent with the results from biotin pulldown in Figs 1c and 3a,b, as well as from TRF2-S RIP in Fig. 2e, where FMRP probably binds and depletes TRF2-S from WT brain lysate, thereby reducing the amount of TRF2-S–RNP complexes.

Taken together, the findings suggested that occupation of the TRF2-S GAR domain by FMRP competitively inhibits RNA binding to the GAR domain of TRF2-S, thereby blocking the TRF2-S–RNP assembly (Fig. 3f).

### TRF2-S RNA-binding activity mediates axonal mRNA transport

**Figure 4:**
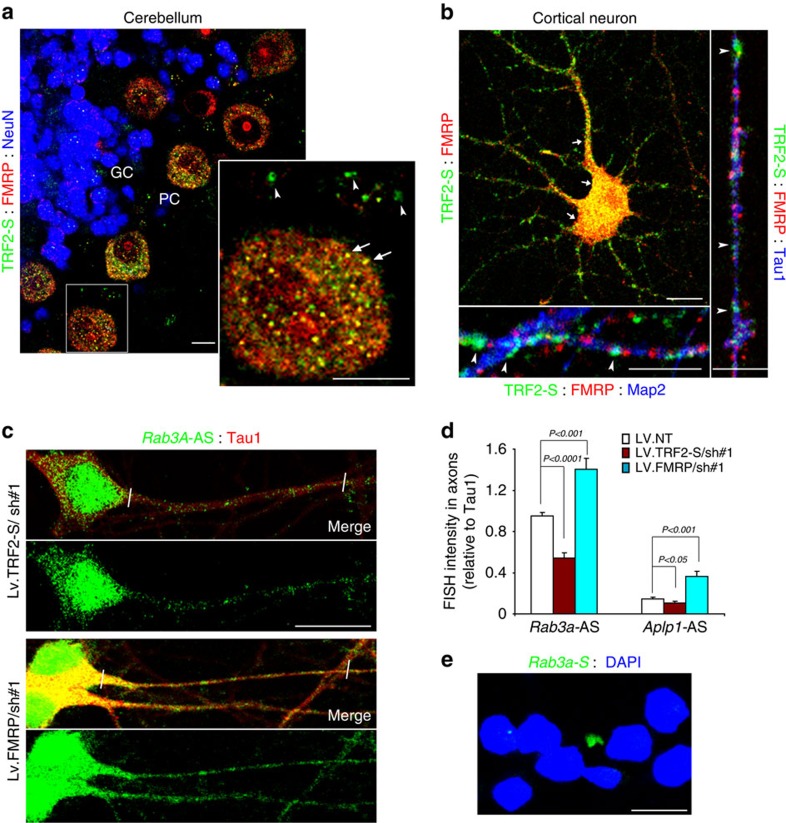
TRF2-S and FMRP are co-localized in the soma but not in the axons. (**a**) Confocal images showing that immunoreactivities of TRF2-S (green) and FMRP (red) were mainly co-localized in the cell body of Purkinje cells (PC) (arrows), whereas FMRP- and TRF2-S puncta (arrowheads) were often apart from each other at outside of PC cell body of rat cerebellum. GC, granule cells; NeuN, a nuclear neural marker; Scale bar, 10 μm. (**b**) Confocal images of cortical neurons (culture day 10) immunostained with TRF2-S (green) and FMRP (red), and co-immunostained either with a dendritic marker MAP2 or an axonal marker Tau1. Contrary to their co-localization in the cell body and proximal dendrites (arrows), TRF2-S and FMRP exhibited little or no co-localization in the axon (arrowheads in right panel) and distal dendrites (arrowheads in bottom panel). Scale bar, 10 μm. (**c**,**d**) On culture day 3 cortical neurons were infected by lentivirus bearing shRNA #1(865) for TRF2-S silencing, shRNA#1(2624) for FMRP silencing and scramble non-target shRNA control. Four days later, cells were fixed for FISH analysis and Tau1 immunostaining (**c**). The fluorescent intensities of *Rab3a* mRNA and Tau1 were measured by 50-μm-long line scan at proximal end of axons (distance between white bars). The bar graph in **d** shows the FISH intensity of *Rab3a* and *Aplp1* mRNAs relative to Tau1 immuoreactivity in axons. *n*=13–16. All values are mean±s.d. *P*-values based on Student's *t*-test. AS, antisense probe; scale bar=20 μm. (**e**) Negative control with a sense (S) probe of *Rab3a* mRNA. Scale bar, 20 μm.

To determine where TRF2-S interacts with FMRP in rat brain tissue and neurons, we performed the double immunostaining of TRF2-S and FMRP. Similar to the fragile X granules being described in the brains^41^, we observed that FMRP and TRF2-S puncta are highly co-localized in the cell body of neurons, whereas in the axons there is relatively little co-localization of TRF2-S and FMRP immunoreactive puncta (Fig. 4a,b). The results suggest that although the TRF2-S GAR domain can bind either to its target mRNAs or to FMRP, there is considerably less FMRP available for inhibition of the formation of TRF2-S–RNP in axons compared with the cell body and dendrites where FMRP is more abundant. We next performed fluorescence *in situ* hybridization (FISH) analysis of the TRF2-S target *Rab3a* and *Aplp1* mRNAs in cultured neurons upon silencing of TRF2-S or FMRP. Results using antisense probes (Fig. 4c,d) and a sense probe control (Fig. 4e) demonstrate that TRF2-S knockdown caused a reduction of *Rab3a* and *Aplp1* mRNA levels in the axons (Fig. 4c,d), whereas FMRP knockdown led to elevated mRNA levels in the axons. However, TRF2-S silencing had little or no effect on the half-life of its target mRNA (Supplementary Fig. 5a–d).

**Figure 5:**
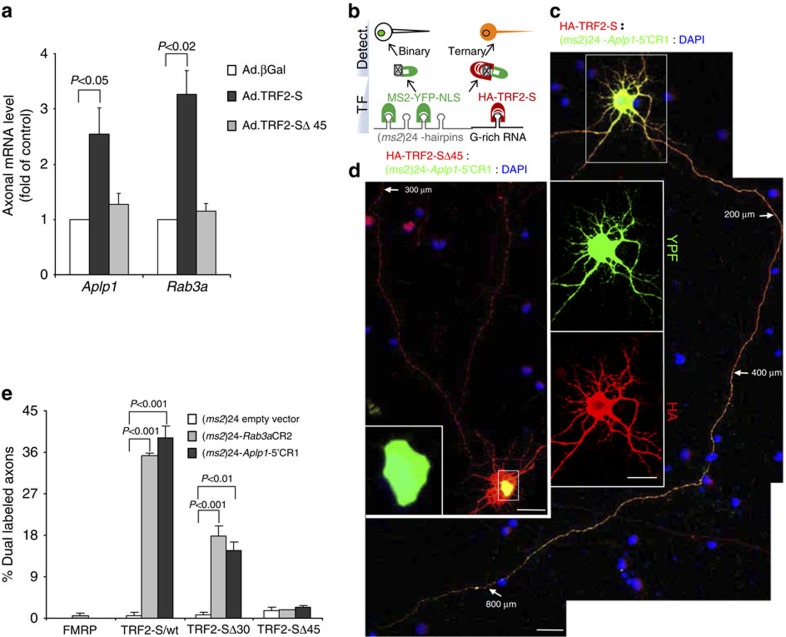
The TRF2-S GAR domain mediates axonal transport of mRNAs. (**a**) RT–qPCR analysis showing that elevating TRF2-S, but not TRF2-S▵45 mutant, significantly increased the mRNA level of *Aplp1* and *Rab3a* in axons relative to βGal control. On culture day 3, neurons were infected for 5 days with adenovirus bearing TRF2-S, TRF2-S▵45 and βGal control, and axonal RNA samples were isolated from the axonal compartments of microfluidic chambers. Values are the fold change normalized to *Gapdh*; *n*=3. (**b**) Schematic of MS2–RNA–HA tracking method. The strategy requires the concomitant expression of *ms2* RNA hairpins (grey loops) tagged TRF2-S target G-rich mRNA (black loop), HA–TRF2-S (red) and MS2–YFP–NLS (green). HA–TRF2-S and MS2–YFP–NLS not only recognize their corresponding RNA motifs, G-rich mRNAs or *ms2* hairpins per se, but also act within the binary or ternary RNP complexes to determine where the RNA chimera is localized within the cell (the nucleus or the cytoplasm). (**c**,**d**) Confocal images show that HA–TRF2-S, but not HA-TRF2-S▵45, is capable of delivering HA- and YFP-labelled ternary RNP complexes into the distal end of axon. Insets are the boxed areas for unmerged images in **c** and nuclear-restricted YFP signal in **d**. On culture day 6, cortical neurons were co-transfected with an appropriate ratio of transgenes for 24 h. Arrows mark distances along the axon from the cell body, scale bars, 10 μm. (**e**) The TRF2-S GAR domain is required for extending the dual-labelled RNP signals into the distal axons (>200 μm). Plasmids HA–TRF2-S, HA–TRF2-S▵30, HA–TRF2-S▵45 and mCherry-tagged FMRP (details in Supplementary Fig. 6d) were examined; each plasmid was co-transfected with MS2–YFP–NLS accompanied either by (*ms2*)24*-Aplp1*-5′CR1 or (*ms2*)24*-Rab3a*-CR2 mRNAs or (*ms2*)24 empty vector control. Fifty transfected neurons per group were examined, *n*=3 separate experiments.

We next asked whether TRF2-S plays a role in transport of mRNAs in the axon. Compartmentalized culture systems have previously proven useful in generating pure preparations of axons^8^,^11^. We chose a high-throughput microfluidic chamber system that consists of two chambers (0.6 × 0.6 cm each) separated by a parallel array of 500-μm-long microgrooves; neurons are plated in one chamber and their axons grow through the microgrooves into the second chamber^42^. In a pilot experiment, we confirmed that cortical neurons cultured in this device for 9 days extended abundant axonal processes, but not dendrites, into the axonal chamber as described previously^42^. This culture system enabled us to obtain a pure preparation of cortical axons characterized by the expression of axonal mRNA *Synaptophysin* (*Syp*), but little or no nuclear mRNA *Histone1* and dendritic mRNA (*CamK2a*) (Supplementary Fig. 6a). We then asked whether introducing TRF2-S or TRF2-S▵45, a mutant lacking the entire RNA-binding GAR domain, would affect the level of *Rab3a* and *Aplp1* mRNAs in axons. RT–qPCR analysis demonstrated that, compared with βGal control, adenoviral induction of TRF2-S but not TRF2-S▵45 significantly increased the axonal level of *Rab3a* and *Aplp1* (Fig. 5a), suggesting that the RNA-binding activity of the TRF2-S GAR domain is indispensable for increasing levels of these mRNAs in axons. We found that TRF2-S elevation had a significant impact on its target mRNAs harvested from the axons but not from the whole-cell lysates (Supplementary Fig. 4a). To determine whether the TRF2-S-induced increase in the level of axonal mRNAs resulted from enhanced axonal entry of the mRNAs, we employed a modified fluorescent RNA (MS2–RNA) tracking system previously used to monitor 3′-untranslated region (UTR) mRNA movements in mammalian cells^43^ and hippocampal neurons^35^. The system included two components: a bacteriophage MS2 protein-fused fluorescent nuclear retention MS2–YFP–NLS and 24 copies of the MS2-binding hairpins (*ms*2)24-tagged 3′-UTR mRNA of interest; for example, *β-actin* 3′-UTR that harbours the intrinsic ‘zipcode' cytoplasmic target sequence enabling the shuttling of yellow fluorescent protein (YFP)-labelled RNP from the nucleus to the cytoplasm^43^. However, in this case TRF2-S-binding sites were mainly located in the coding regions of *Rab3a* and *Aplp1* mRNAs, which lacked ‘zipcode' sequences and thus they were incapable of shuttling MS2–YFP–NLS-labelled RNP out of the nucleus (Supplementary Fig. 6c). To visualize axonal translocation of mRNA, we included a third transgene, HA–TRF2-S, in the system. As illustrated in Fig. 5b, when MS2–YFP–NLS binds to the *ms2* RNA motifs within (*ms2*)24-G-rich mRNA chimera, a YFP-labelled binary RNP complex resides in the nucleus by default, unless HA–TRF2-S also simultaneously binds to the G-rich motif in the RNA chimera. The latter interaction enables the translocation of a YFP– and HA–TRF2-S-associated RNP ternary complex from the nucleus into the axon due to the presence of the RNA-binding GAR domain and a nuclear export signal in TRF2-S (Fig. 1a)^30^. To validate that this system functions in intact cells, we performed a pilot experiment and found that overexpression of either MS2–GST (replacing MS2–YFP–NLS) or HA–TRF2-S enabled the pulldown of an *ms2*-tagged G-rich RNA chimera (Supplementary Fig. 6b).

To establish the efficiency of YFP–/HA–labelled RNP ternary associations, cortical neurons were co-transfected with a 3:1.5:1 ratio of (*ms2*)24-tagged axonal G-rich mRNAs: HA–TRF2-S:MS2–YFP–NLS. As shown in Fig. 5c,e, when HA–TRF2-S was co-expressed with MS2–YFP–NLS and (*ms2*)24–RNAs, the double fluorescence-labelled RNP signal was no longer retained in the nucleus, but rather extended robustly into the axons, travelling over 800 μm from the soma to the distal end of the axon. In contrast, when using HA–TRF2-S▵45 to replace HA–TRF2-S in the same system, little or no YFP-labelled RNP signal was shuttled into the axons. Instead, the majority of signal was retained in the nucleus (Fig. 5d,e). The latter result is consistent with an RNA-trap assay where HA-▵45 failed to precipitate *ms2*-tagged *Aplp1* RNA (Supplementary Fig. 6b). Collectively, these results suggest that the RNA-binding activity of TRF2-S GAR domain is critical for the translocation of TRF2-S target mRNAs into axons.

### FMRP inhibits axonal translocation of TRF2-S-bound RNAs

To begin to elucidate the roles of TRF2-S and FMRP during neuronal development, we measured the expression patterns of endogenous TRF2-S and FMRP during the course of neuronal maturation. We found that TRF2-S expression increased gradually and was accompanied by the upregulation of the synaptic markers synaptophysin and PSD95 as neurons matured, whereas FMRP expression declined during the period of active synaptogenesis (Supplementary Fig. 7a).

**Figure 6:**
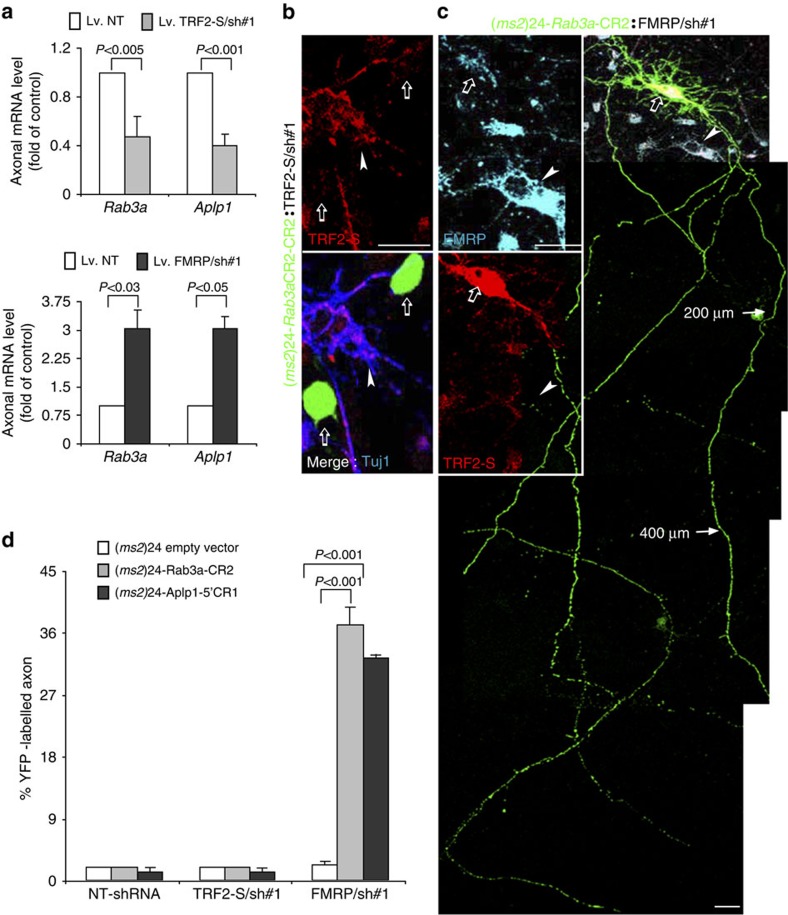
FMRP inhibits the transport of TRF2-S target mRNAs into axons. (**a**) RT–qPCR was used to assess whether silencing of endogenous TRF2-S (upper bar graph) or FMRP (lower bar graph) affected the axonal mRNA levels. Neurons grown in microfluidic chambers were infected on culture day 3 with lentivirus bearing shRNA #1(865) for TRF2-S silencing, shRNA#1(2624) for FMRP silencing and scramble non-target shRNA control, and 5 days later axonal mRNAs were isolated. Values represent the fold change normalized to *Gapdh*; *n*=3. (**b**,**c**) Representative confocal images show that silencing of FMRP, but not TRF2-S, induced the translocation of YFP-labelled RNP complexes into axons. On culture day 6, neurons were co-transfected with an appropriate ratio of MS2–YFP–NLS and (*ms2*)24-*Rab3a*CR2 RNA in combination with shRNA vectors for silencing of TRF2-S/sh#1 (865) or FMRP/sh#1(2624) for 52 h. The open arrows in **b** point to YFP-labelled RNP signals restricted in the nuclei of transfected neurons where TRF2-S immunoreactivities were scarce as compared with naive cells (arrow head). An open arrow in **c** points to the YFP-labelled RNP signals extended in the axons of a transfected neuron where FMRP expression was reduced as the comparison with a naive cell (arrow head). Arrows mark distances along the axon from the cell body. Tuj1, a neuronal marker. Scale bar, 20 μm. (**d**) Quantification showing that percentages of FMRP/shRNA#1- and TRF2-S/shRNA#1-transfected neurons that exhibited the YFP-labelled RNP complexes in the distal axons (>200 μm). Fifty neurons were evaluated per group, *n*=3 separate experiments.

We next examined the consequences of selective reduction of TRF2-S and FMRP levels on axonal mRNA expression. In pilot experiments, we examined the gene knockdown efficiency of two sets of short hairpin RNAs (shRNAs) previously used for silencing of TRF2-S^30^ and FMRP^44^ in rat neurons (Supplementary Fig. 7b,c). TRF2-S knockdown resulted in a significant downregulation of its target axonal mRNAs, *Aplp1* and *Rab3a*, in isolated axons (Fig. 6a) and whole-cell lysates (Supplementary Fig. 4b). In contrast, knockdown of FMRP promoted axonal expression of these mRNAs (Fig. 6a). Consistently, knockdown of FMRP, but not TRF2-S, significantly increased the translocation of YFP-labelled RNP complexes into the axons (Fig. 6b–d). As an additional validation, we found that overexpression of mCherry-tagged FMRP completely failed to counteract the nuclear retention of the fluorescent RNA signal (Fig. 5e and Supplementary Fig. 6d).

Taken together with the evidence that FMRP binds to the TRF2-S GAR domain rather than to TRF2-S mRNA ligands, these results from TRF2-S knockdown experiments suggest that TRF2-S plays a pivotal role for selectively regulating its target mRNAs in axons, whereas FMRP inhibits the axonal translocation of TRF2-S–RNPs probably by retarding the assembly of TRF2-S–RNP complexes.

### TRF2-S and FMRP differentially regulate axonal growth

**Figure 7:**
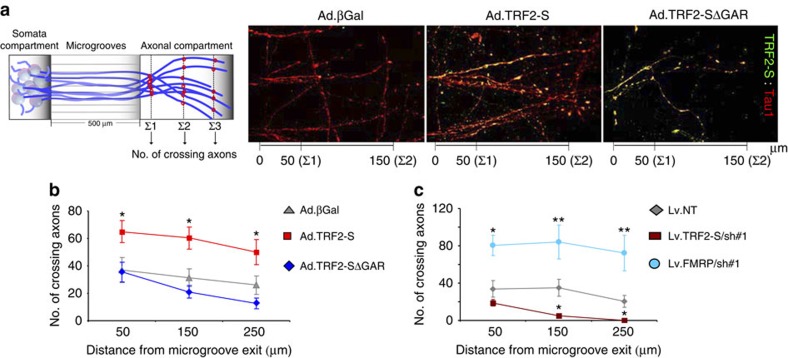
TRF2-S promotes and FMRP inhibits axonal growth. (**a**) Left, illustration of a microfluidic chamber and analysis of outgrowth in the axonal compartment. ∑1, ∑2 and ∑3 indicate three different distances within the axon compartment used to quantify the number of axon crossings (red dots). Right, cortical cultures (3 days in culture) in the microfluidic chamber AX500 were infected by adenovirus bearing βGal, TRF2-S and TRF2-S▵45 for 4 days. Axons and the level of TRF2-S were visualized by coimmunostaining with Tau1 (red) and TRF2-S (green) antibodies. (**b**) On culture day 3, cortical cultures in AX500 chambers were infected with adenovirus bearing βGal, TRF2-S or TRF2-S▵45. Four days later, axon outgrowth was measured by counting the number of crossing axons at 50, 150 and 250 μm in the axonal compartment. A total of 24 images from two separate experiments were analysed by two-way analysis of variance following Bonferroni's post test (*P<0.05). (**c**) On culture day 3, neurons were infected with lentivirus carrying a non-target shRNA(NT) and target-specific shRNAs for silencing of TRF2-S/sh#1 (865) or FMRP/sh#1(2624); 5 days later axonal outgrowth was analysed as described in **b** (*P<0.05, **P<0.01). *n*=4 separate experiments.

We next determined whether manipulation of TRF2-S and FMRP levels affects axonal growth. We employed another microfluidic multi-chamber system (AX500), the design of which enables growth of a low density of axons, suitable for quantifying axon outgrowth (Fig. 7a). Neurons were infected on culture day 3 with adenovirus bearing TRF2-S, TRF2-S▵GAR or βGal, and 4 days later the expression levels within the axons were examined by co-immunostaining of TRF2-S and Tau1. We counted the number of axons that extended 50, 150 and 250 μm into the axon compartment as illustrated in Fig. 7a. TRF2-S overexpression significantly enhanced axon outgrowth compared with neurons overexpressing TRF2-S▵GAR or βGal (Fig. 7b).

We also examined the effects of TRF2-S and FMRP knockdown using an AX500 microfluidic system. On culture day 3, neurons were infected with lentivirus carrying the scrambled shRNA as the non-target control, TRF2-S shRNA or FMRP shRNA, and axon lengths were quantified 5 days later. Compared with neurons expressing the non-target shRNA, TRF2-S knockdown remarkably reduced the length of axons, whereas FMRP knockdown significantly increased the length of axons (Fig. 7c).

### TRF2-S and FMRP differentially affect presynaptic function

**Figure 8:**
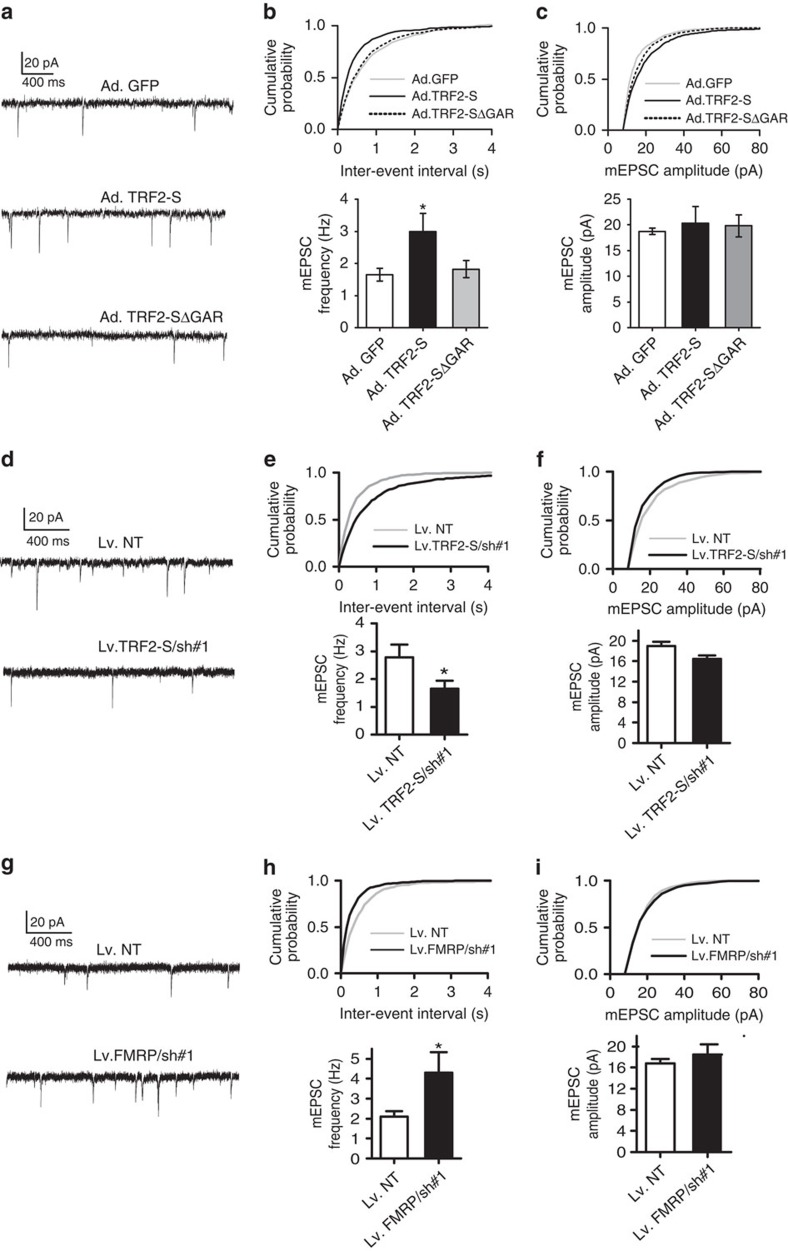
TRF2-S and FMRP differentially affect synaptic glutamate release. (**a**–**c**) On culture day 6, hippocampal neurons were infected with Ad.GFP, Ad.TRF2-S or Ad.TRF2-S▵45. The mEPSCs were measured on culture day 15. Representative mEPSC traces from transduced neurons are shown in **a** and quantification of the frequency and amplitude of mEPSCs is shown in **b**,**c**. The frequency of mEPSCs in neurons expressing TRF2-S was significantly greater than those expressing βGal or the TRF2-S▵45 mutant (*P<0.01). (**d**–**i**) mEPSCs were measured in hippocampal neurons on culture day 15, which was 7 days after lentiviral transduction with a non-target (NT, scramble) shRNA and target-specific shRNAs for silencing of TRF2-S/sh#1(865) or FMRP/sh#1 (2624). Representative mEPSC traces are shown in **d**,**g**. Quantification of the frequency and amplitude of mEPSCs is shown in (**e**,**f**,**h**,**i**) (*P<0.05). *n*=15 neurons per condition.

As two mRNAs to which TRF2-S binds encode Rab3a and Aplp1, which are known to play roles in regulating neurotransmitter release^37^,^38^, we determined whether TRF2-S influences neurotransmitter release. We examined synaptic transmission in cultured hippocampal neurons. On culture day 5, hippocampal neurons were infected with adenovirus bearing TRF2-S, TRF2-S▵GAR or GFP, and 10 days later miniature excitatory postsynaptic currents (mEPSCs) mediated by the neurotransmitter glutamate were recorded. TRF2-S expression caused a significant increase in mean mEPSC frequency compared with neurons overexpressing Ad.GFP and Ad.TRF2-S▵GAR (Ad.GFP, 1.65±0.025; Ad.TRF2-S, 2.98±0.0561; and Ad.TRF2-S▵GAR, 1.823±0.268 Hz; *P*<0.01; *n*=12 per group; Fig. 8a,b). There was no significant effect of TRF2-S transduction on the amplitude of mEPSCs (Fig. 8c). The results indicated that RNA-binding activity of TRF2-S was necessary for enhancing presynaptic function.

We next determined the effects of silencing TRF2-S or FMRP on synaptic plasticity. On culture day 5, hippocampal neurons were infected with lentiviral shRNAs specific for silencing TRF2-S (TRF2-S shRNA#1(865)) or FMRP (FMRP shRNA#1(2624)) and electrophysiological recordings were performed 10 days later. Compared with non-target shRNA controls, silencing TRF2-S resulted in a significant decrease in mean mEPSC frequency (non-target (NT) control, 2.78±0.45; TRF2-S/shRNA#1, 1.64±0.29 Hz; *P*<0.05; *n*=15 per group; Fig. 8d,e), whereas silencing FMRP resulted in a significant increase of the mEPSC frequency (NT control, 2.1±0.025; FMRP/shRNA#1, 4.3±1.02 Hz; *P*<0.05; *n*=15 per group; Fig. 8g,h). The amplitude of mEPSCs was not significantly affected by knockdown of either TRF2-S (Fig. 8f) or FMRP (Fig. 8i). Taken together, these findings suggest that TRF2s enhances release of glutamate from presynaptic terminals, whereas FMRP inhibits glutamate release.

## Discussion

Our findings reveal that TRF2-S is a novel RBP critical for axonal mRNA transport. Multiple lines of evidence indicate that the GAR domain of TRF2-S is necessary for its binding either to FMRP or to target RNAs. Occupancy of TRF2-S by FMRP inhibits the binding of TRF2-S to its mRNA ligands. As neurons mature and form synapses during neural development, TRF2-S levels increase, while FMRP levels decrease, and TRF2-S assumes important roles in axonal plasticity. We found that overexpression of TRF2-S and silencing of FMRP promote axonal mRNA transport, which is correlated with axon outgrowth and increased excitatory neurotransmitter (glutamate) release from presynaptic terminals. Our findings suggest a pivotal role for TRF2-S in an axonal mRNA localization pathway that enables local protein synthesis by counteracting FMRP-mediated inhibition, thereby promoting axonal outgrowth and enhancing neurotransmitter release.

Several methods have been previously used to identify global *in vivo* RNA–protein interactions including immunopurification of RBP followed by microarray analysis (RIP-Chip) and covalent ultraviolet cross-linking followed by IP and high-throughput RNA sequencing (CLIP). In comparison, the major advantage of CLIP is to identify individual binding sites of RBP within an RNA target^32^. However, owing to technical difficulties we were unable to apply *in vivo* CLIP to primary cultured neurons. Nevertheless, by using a combination of *in vivo* RIP-Chip with a modified *in vitro* CLIP, we confirmed that TRF2-S is a neural specific RBP with a single RNA-binding GAR domain that directly associates with a subset of neuronal mRNAs. The latter findings are consistent with a previous report that the GAR domain in nuclear TRF2 is necessary for binding of telomere-associated RNA^27^. Upon covalent cross-linking of GST–TRF2-S with a segment of its cognate (*Trf2-S*) mRNA, we were able to map the TRF2-S–mRNA-binding sequences in *Trf2-S*. Importantly, the TRF2-S GAR domain is indispensable for assembling the G-rich *Aplp1* and *Rab3a* mRNA complexes. A previous study reported that G-rich RNA sequences or G-quartets act as specific RNA motifs recognized by GAR/RGG domain-containing RBPs and thus serves as a signal for mRNA targeting in neurites^45^. Consistent with this notion, we found that the binding of the TRF2-S GAR domain to these G-rich mRNA fragments enables the shuttling of the fluorescent RNA reporter complex from the nucleus into the distal end of axons, indicating that the RNA-binding capability of GAR domain in TRF2-S is essential for facilitating the entry of target mRNAs into axons.

In addition to *Rab3a* and *Aplp1*, two axonal mRNAs, the gene list from the profiling of the TRF2-S-associated transcriptome and RT–qPCR analysis identified a subgroup of TRF2-S target transcripts that also encode axonal proteins. By comparison with two published axonal cDNA libraries from the pure preparations of cortical axons^8^ and sensory axons^11^, ∼24% of the 140 putative TRF2-S target transcripts we identified in our search overlapped with the axonal transcriptomes (Supplementary Table 2). Where and how TRF2-S bound to this larger subset of mRNAs and its impact on axonal physiology remains to be determined.

Interestingly, previous findings suggest that full-length telomeric TRF2 can interact with the FMRP-associated proteins FXR1, FXR2 and CYFIP1 either directly^46^ or via TERRA-RNA^27^,^47^ in proliferative non-neuronal cells. It will therefore be of considerable interest to elucidate the roles of the nuclear telomeric DNA- and RBP TRF2, and the cytoplasmic RBP TRF2-S, as neural stem cells cease dividing and differentiate into neurons.

As FMRP regulates the translation and localization of dendritic mRNAs via its RNA-binding activity^33^,^48^,^49^,^50^, we determined whether FMRP also bound to TRF2-S target mRNAs. In contrast to expectation, we were unable to detect direct interactions of FMRP with three TRF2-S-bound transcripts, namely *Trf2-S*, *Rab3a* and *Aplp1* mRNAs. However, FMRP derived from brain lysate was readily detectable within TRF2-S–RNA complexes. Given that high-salt buffers are commonly used for promoting protein–protein interaction by release of proteins from RNP complexes^36^, an explanation for later result is that a high-salt (450 mM KCl) Co-IP buffer was used for homogenizing brain tissue before biotin pulldown, resulting in the preoccupation of FMRP with the GAR domain of TRF2-S, thereby preventing the formation of TRF2-S–RNA complexes *in vitro*. Indeed, using an RNA mobility-shift assay we found that FMRP inhibits the binding of TRF2-S to its target mRNA.

The ‘mGluR theory'of fragile X syndrome^21^ is focused on the postsynaptic function of FMRP in regulating dendritic translation stimulated by mGluR5 activation^45^,^49^. However, although dysregulation of dendritic protein synthesis probably contributes to the impaired cognitive function in Fragile X syndrome (FXS), the mechanisms underlying axonal abnormalities in this disorder are poorly understood. FMRP-containing RNP granules are found in axons and presynaptic terminals^23^,^24^,^41^, and several putative presynaptic FMRP target transcripts have been identified from analysis of the mRNA translational profile in Fmr1 KO mice^18^,^19^. In addition, recent studies of Fmr1 KO or Fmr1 mosaic KO mice have provided evidence for cell-autonomous presynaptic functional abnormalities^22^,^51^. Two independent studies recently demonstrate that FMRP can regulate neurotransmitter release in hippocampal and cortical pyramidal neurons by a mechanism involving an interaction of FMRP with Ca^2+^ activated K^+^ (BK) channels^22^ and the modulation of N-type Ca^2+^ channel^52^; loss of FMRP-mediated action potential broadening was rescued with an FMRP N-terminal protein-binding peptide but not with protein translation inhibitors^22^, suggesting a translation-independent role of FMRP for presynaptic functions.

Our results suggest a protein binding-based FMRP function in regulating axonal/presynaptic plasticity, a mechanism that differs from the current FMRP–mRNA interaction model for regulating dendritic mRNA translation. Our mutational and Co-IP analyses suggest the GAR domain in TRF2-S is required for binding to FMRP and to mRNA ligands, perhaps in a mutually exclusive manner. Under physiological low-salt conditions, TRF2-S binds to axonal mRNAs, whereas removal of RNA either via RNase treatment or by a high-salt buffer permits TRF2-S binding to FMRP. Furthermore, by occupying the GAR domain of TRF2-S, FMRP can competitively inhibit the assembly of TRF2-S–mRNA complexes. Importantly, in intact neurons we observed that HA–TRF2-S, but not mCherry–FMRP, was co-localized with MS2–YFP-tagged TRF2-S target RNAs in axons. Thus, in addition to biochemical evidence the latter results also suggested that TRF2-S, but not FMRP, bound to its target mRNAs in axons, to enhance the transport of axonal mRNAs.

RNA localization-based mechanisms may couple extrinsic signals to local cellular responses in processes such as synapse formation, neurite outgrowth and retraction, axon guidance, injury-induced axonal regeneration and activity-dependent synaptic plasticity^3^. We found that elevation of TRF2-S enhanced axon outgrowth and EPSCs at excitatory hippocampal neuron synapses, suggesting that TRF2-S plays a positive role in axon elongation and the release of glutamate from presynaptic terminals. The enhancement of axon growth and neurotransmitter release by TRF2-S is dependent on its GAR domain, consistent with the axonal mRNA-binding function of TRF2-S in the regulation of axonal plasticity. We found that TRF2-S targets several mRNAs that encode proteins known to control axonal transport and synaptic vesicle dynamics including *Rab3a* and *Aplp1* (refs 37, 38). Such mRNA targets are likely to be involved in the mechanism by which TRF2-S regulates axonal plasticity. It remains to be determined whether TRF2-S knockdown decreases the amount of glutamate released from individual synapses.

It has been reported that FMRP inhibits axon growth^21^ and glutamate release^22^, possibly by modulating action potential duration through a translation-independent effect of FMRP. Similarly, we found that knockdown of FMRP resulted in enhanced axon outgrowth and an increased EPSC frequency at glutamatergic synapses of hippocampal neurons. Our findings suggest that the impact of FMRP silencing on axonal plasticity may not be through translational control by FMRP, but rather by unmasking the RNA-binding activity of TRF2-S, thereby enabling the transport of axonal mRNAs. Although our findings provide evidence that TRF2-S interacts with FMRP, and that this interaction influences axon outgrowth and neurotransmitter release, the data do not allow a definitive conclusion as to whether FMRP–TRF2-S interactions are axon specific. Indeed, as FMRP has been shown to regulate translation of several mRNAs in the neuronal soma and dendrites, effects of FMRP–TRF2-S interactions on axonal mRNA localization and axonal plasticity could be a consequence of global changes in mRNA regulation^53^.

Loss of FMRP in fragile X syndrome leads to dysregulation of its target transcripts, which in turn alters synaptic development and function, and impairs long-term memory^48^,^54^. Interestingly, loss of presynaptic FMRP had no effect on excitatory synapses onto excitatory neurons but did reduce glutamate release onto fast-spiking inhibitory cortical neurons^55^. We found that TRF2-S is in particular abundant in projection neurons and is expressed in much lower amounts in intrinsic neurons, and it will therefore be important to establish the roles for TRF2-S and FMRP in different subpopulations of neurons and synapses.

## Methods

### Animals

Fmr1 KO (B6.129P2-*Fmr1*^*tm1Cgr*^/J; stock # 003025) and WT control (C57BL/6 J; stock #000664) mice were obtained from the Jackson Laboratories, and were propagated in our facility. Genotyping was performed according to the Jackson Laboratories protocols. Data from *Fmr1* KO female mice were compared with age-matched WT female mice. Mice were deeply anaesthetized with isoflurane before decapitation. Timed pregnant female Sprague–Dawley rats were purchased from Charles River Laboratories and were used as source of embryos for primary neuronal culture. Some pregnant rats were allowed to deliver pups, which were then used as a source of adult brain tissue when they were 2 months old. All procedures were approved by the Animal Care and Use Committee of the National Institute on Aging Intramural Research Program.

### Cell culture and gene delivery

HEK-293FT and HEK-293 A cells (Invitrogen) were grown in DMEM medium (Invitrogen) supplemented with 10% fetal clone III (HyClone). Cerebral cortical and hippocampal neurons isolated from 18-day-old rat embryos were plated at a density of 2–6 × 10^5^ cells per well as described^56^.

For cell imaging studies, neurons were cultured on polyethyleneimine-coated 18-mm glass coverslip for 5 days and then were subjected to Lipofectamine 2000 (Invitrogen)-mediated transfection with 1.75 μg of cDNA mixture in OptiMEM medium. After incubation for 2 h, cells were washed once and the medium replaced with neuron-conditioned Neurobasal-B27 medium (Invitrogen).

For axon outgrowth analysis, neurons were cultured in microfluidic chambers AX500 (Millipore) with a microgroove length of 500 μm, following the manufacturer's instructions. Briefly, AX500 chambers were sterilized with 70% ethanol and then coated with 0.5 mg ml^−1^ poly-D-lysine in 50 mM sodium borate solution. Six microlitres of neuronal cell suspension (∼5–10 × 10^6^ cells per ml) was plated at the opening of the cell body compartment. After cell attachment, 200 μl of fresh Neurobasal medium with B27 supplement (ThermoFisher Scientific) was placed in each well. Seventy-two hours after plating, neurons were infected with 1 μl of purified lenti-shRNA virus or adenovirus for 6–12 h. Virus inoculation was removed and replaced with neuron-conditioned Neurobasal-B27 medium. Similar viral inoculating procedures were applied for large-scale neuronal cultures in 60-mm plates.

For analysis of axonal mRNAs, ∼4.5 × 10^4^ cortical neurons were plated in cell body compartments of a poly-D-lysine-coated microfluidic (96-well high-throughput format) chamber^42^ with a microgroove length of 500 μm, to allow robust axon extensions into an axonal compartment. After 9 days, the microfluidic chambers were washed two times with ice-cold PBS. After thoroughly removing cellular contexts from the cell body compartment by aspiration, 100 μl of TRIzol reagent (Invitrogen) was added immediately into the axonal compartment and the solution containing axonal RNA was triturated mildly. Isolated RNA from three axonal compartments was pooled.

To target rat TRF2-S (GenBank accession: NM_001242355.1), five gene-specific oligonucleotides (21-mer) were selected for the construction of small interfering hairpins and cloned into pLKO.1 lentiviral vector as described previously^30^. To target rat FMRP (GenBank accession: NM_052804.1), five gene-specific lenti-shRNAs in pLKO.1 vector were purchased from the RNAi Consortium (TRC) Lentiviral shRNA Library (Openbiosystem). After preliminary testing, we chose two shRNAs that have been tested in previous studies for silencing TRF2-S—shRNA-774 (5′- GCACACAGAGCCAGTGGAGAA -3′) and shRNA-865 (5′- GCTTTCAAAGCTCTGTCTACT -3′)^30^, and two shRNAs for silencing FMRP—shRNA2623 (5′- CCACCACCAAATCGTACAGAT -3′, Clone ID:TRCN0000102623) and shRNA 2624 (5′- GAGGATGATAAAGGGTGAGTT -3′, Clone ID:TRCN0000102624)^44^. The lentivirus particles were produced by co-transfecting shRNA/pLKO.1 vector with psPAX2 and pMD2.G (Addgene) into HEK-293T cells according to the protocol provided by Addgene. Briefly, the viral particles from culture media were harvested at 36 and 48 h post transfection and concentrated using a Sorvall TH-641 swinging bucket rotor at 26,000 r.p.m. for 2 h. The virus pellet was suspended in sterile PBS with 1% BSA at a density of ∼6 × 10^7^ virions per ml.

For transduction of neurons with adenovirus carrying GFP, βGal, TRF2-S and TRF2-S ΔGAR mutant were packaged into 293A cells as described previously^30^. Adenoviral particles were purified using a Vivapure AdenoPACK 100 kit (Sartorius AG, Goettingen, Germany) according to the manufacturer's directions.

### Plasmids and protein purification

WT TRF2-S and TRF2-S mutants were generated by PCR using the Phusion DNA polymerase (New England Biolabs) and touch-down PCR programme as described previously^30^. PCR products were subcloned into HA-tag, GST-tag and eGFP-tag vectors. GST–mFMRP and GST–mFMRP-ct plasmids were generously provided by Dr Hye Young Lee^35^.

For generating GST-tagged recombinant proteins, BL21 (*Escherichia coli*) cells were transformed with individual expression vectors encoding the GST fusion proteins and lysed in buffer A consisting of 50 mM Tris-HCl pH 7.6, 150 mM NaCl, 2 mM EDTA, 10% glycerol, 1% Triton X-100, 2 mM phenylmethylsulfonyl fluoride, 1 mM dithiothreitol (DTT), 1% sacrosine and a protease inhibitor cocktail (Roche). After clearing lysates by centrifugation at 12,000*g* for 30 min at 4 °C, the recombinant proteins were purified with glutathione-Sepharose 4B.

### RIP and microarray analysis

Endogenous mRNA–protein complexes were immunoprecipitated (RIP) as described previously^29^. Briefly, the high-density cortical cultures (∼1.2 × 10^8^ neurons) and freshly dissected mouse cerebella were homogenized in polysome lysis buffer consisting of 100 mM KCl, 5 mM MgCl_2_, 10 mM HEPES pH 7.0, 0.5% Nonidet P-40, 1 mM DTT, 100 U ml^−1^ RNase OUT (Invitrogen) and a protease inhibitor cocktail (Roche). Lysates were incubated (1 h at 4 °C) with 100 ml of a 50% (v/v) suspension of protein-A Sepharose beads precoated with 20 μg each of polyclonal anti-TRF2-S (Santa Cruz, H300) or rabbit IgG. Beads were washed with NT2 buffer consisting of 50 mM Tris-HCl (pH 7.4), 150 mM NaCl, 1 mM MgCl_2_ and 0.05% NP-40, and then incubated with 100 ml of NT2 buffer containing RNase-free DNase I (20 U, 15 min at 30 °C), washed with NT2 buffer and further incubated in 100 ml NT2 buffer containing 0.1% SDS and 0.5 mg ml^−1^ Proteinase K (15 min at 55 °C), to digest proteins bound to the beads. RNA was extracted using phenol and chloroform, precipitated in the presence of glycoblue (Applied Biosystems) and used for further analysis.

For Illumina microarray analysis, the RNA obtained after RIP reactions using either anti-TRF2-S or IgG antibodies was assessed using an Agilent 2,100 bioanalyser and RNA 6,000 nanochips. The RNA was used to generate biotin-labelled RNA using the Illumina Total Prep RNA Amplification Kit (Ambion), which was then hybridized to Sentrix RAT ref-12 Expression BeadChips (Illumina, San Diego, CA), containing ∼22,000 well-annotated RefSeq transcripts with ∼30-fold redundancy. The arrays were scanned using an Illumina BeadStation 500X Genetic Analysis Systems scanner and the image data extracted using Illumina BeadStudio software, version 1.5, normalized by *Z*-score transformation and used to calculate differences in signal intensities. Significant values were calculated from two groups of independent experiments, using a two-tailed *Z*-test with *P*<0.05, a false discovery rate <0.30, a *z*-ratio absolute value not <1.5 and an average signal intensity not <zero. The results also had to pass the filtering and one-way independent analysis of variance test by sample groups <0.05 and detection *P*-value for any probe in the comparison group <0.02.

### RT–qPCR biotin pulldown assay and *in vitro* mapping of binding site for RNA–protein interaction

Total RNA was isolated from cells using TRIzol (Invitrogen) from intact cells or from RIP samples and was used to measure gene expression or to validate microarrays, respectively. After RT using random hexamers or oligo dT primers and SSII reverse transcriptase (Invitrogen), real-time qPCR analysis was performed using gene-specific primer pairs and SYBR Green PCR master mix (Kapa Biosystems).

For biotin pulldown assays, brain cytoplasmic lysates or purified GST-tagged proteins were prepared in buffer B consisting of 20 mM HEPES-KOH (pH 7.5), 25% glycerol, 0.1 mM EDTA, 5 mM MgCl_2_, 0.25% NP-40, 450 mM KCl, 1 mM DTT and a protein inhibitor cocktail (Roche). PCR fragments containing the T7 RNA polymerase promoter sequence ((T7) 5′- CCAAGCTTCTAATACGACTCACTATAGGGAGA -3′) were used as templates for *in vitro* transcription. The oligomer pairs used for PCR amplification of the RNA fragments are listed in Supplementar Table 3. Biotinylated transcripts (0.5–5 μg) were incubated either with 450 μg of protein of brain cytoplasmic lysates or with 2 μg of recombinant purified protein (GST, GST–TRF2-S, GST–FMRP and GST–FMRP-ct) for 30 min at room temperature with reaction buffer containing final concentration of 120 mM KCl. The complexes were isolated with streptavidin-coated magnetic Dynabeads (Dynal) and subjected to immunoblot analysis.

To map binding sites of TRF2-S on *Trf2-S* mRNA *in vitro*, 10 μg of GST–TRF2 recombinant protein and GST control protein purified from *E. coli* were incubated with 5 μg of *Trf2-S* mRNA fragment (CR1, 500 nt) transcribed *in vitro* in 1 ml of PEB buffer containing 20 mM Tris-HCl pH 7.5, 100 mM KCl, 5 mM MgCl_2_ and 0.5% NP-40 at 4 °C for 2 h. Upon ultraviolet cross-linking with 150 mJ radiation (245 nm wave length), the RNA–protein complex was treated with 10 U μl^−1^ of RNase T1 for 1 h at 25 °C for removal of unbound RNAs, followed by purification of GST pulldown. On incubation with GST agarose beads at 4 °C for 2 h, the pellet with bound RNA was washed with PEB three times and incubated with 2 μg μl^−1^ Proteinase K at 55 °C for 30 min. The small RNA fragments were recovered by phenol/chloroform extraction followed by ethanol precipitation. After assessing the quality and quantity, the small RNAs were ligated to 5′- and 3′-adaptors before reverse transcription and PCR amplification using Ion total RNA-seq kit v2 as described in the manufacturer's instructions (Life technologies), and were then subjected to T–A cloning. To enhance the specific signal of the insert, rolling circle amplification was performed before DNA sequencing.

### Immunoblot and RNA electrophoretic mobility-shift assays

Adult rat brain tissue samples and HEK293A cells were lysed in buffer B (as described in the previous section) and subjected to Co-IP analysis. For brain lysates, the cytoplasmic fractions were pre-cleared with protein A/G plus-agarose beads (Santa Cruz), then immunoprecipitated with 5 μg of TRF2 (N-20) (Santa Cruz, sc-9528X) and 2 μg of FMRP (7G1-1) concentrated hybridoma supernatant (the hybridoma bank at the University of Iowa) antibodies. For HA–IP, cell lysates were precipitated with the HA-affinity matrix (Roche). After high stringency washes with buffer B containing 300 mM KCl, bound proteins were analysed by immunoblotting. Proteins for immunoblotting were transferred electrophoretically to a polyvinylidene difluoride membrane (Bio-Rad), which was then incubated in blocking solution (5% milk powder in Tween tris buffered saline (TTBS)) for 1 h and then incubated overnight at 4 °C in the presence of antibodies against the following: TRF2-S (H300) and FMRP (H120) (Santa Cruz) with 1:300 dilution, GFP (Covance) with 1:5,000 dilution, HA (Roche) with 1:2,000 dilution, Rab3a (Synaptic Systems) with 1:1,000 dilution and β-actin (Sigma) with 1:10,000 dilution. eukaryotic elongation factor 2 polyclonal antibody (1:1,000 dilution) was kindly provided by Dr. Antonio Ayala at the University of Seville, Spain. After washes in TTBS, the membrane was incubated for 1 h in the presence of the species-appropriate peroxidase-conjugated secondary antibody (Jackson Immunoresearch) and then washed in TTBS. Immunolabelled proteins were visualized using an enhanced chemiluminescence kit (Amersham) or Femto-Supersignal kit (Pierce). To reprobe blots with multiple antibodies, the membrane was stripped using the Restore Western blot stripping buffer (Pierce). The full-scan images of immunoblots are shown in Supplementary Figs 8–14.

Lysates from HEK 293 A cells expressing WT HA-TRF2-S was mixed with purified GST–FMRP (15 μg per reaction) before HA-affinity purification. The precipitates resulting from HA-affinity pulldown were washed four times with buffer B with 450 mM KCl and twice with RNA binding buffer (10 mM Tris-HCl pH 8.0, 100 mM KCl, 0.3 mM MgCl, 10 mM DTT, 5 mM phenylmethylsulfonyl fluoride). Two micrograms of of biotinylated RNA probes and 200 U RNaseOut were incubated with each reaction at room temperature for 1 h with rotation. The shifted protein–RNA complexes were separated by electrophoresis in a 0.7% agarose gel and stained with SYBR green II RNA stain solution (Molecular Probes) in dH2O (1:5,000 dilution) for 20 min.

### Immunocytochemistry and FISH

In most cases, neurons were fixed by incubation in 4% paraformaldehyde in PBS for 35 min. For Tau1 immunostaining, neurons were sequentially fixed with 4% paraformaldehyde and cold methanol at −20 °C for 10 min. Following PBS washes, fixed cells were permeabilized with 0.2% Triton X-100 in PBS for 10 min, then incubated with blocking buffer (3% BSA, 5% normal serum in PBS) for 1 h and then incubated with combinations of primary antibodies in blocking buffer overnight at 4 °C. The primary antibodies used were: TRF2 (Clone 4A794.15, Imagenex) with 1:100 dilution and Tau1 (Clone PC1C6, Chemicon) with 1:200 dilution. After thorough washing, cells were incubated with 1:400 dilution of Alexa 488-, Alexa 568- and Alexa 647-conjugated secondary antibodies appropriate for the specific primary antibodies, followed by 4,6-diamidino-2-phenylindole nuclear counter stain. The cells were examined and images acquired using a Zeiss LSM510 confocal laser-scanning microscope with × 63 water- or × 40 oil-immersion objectives.

FISH analysis was performed using a method described by Lee *et al*.^35^ and the Singer Lab (http://www.singerlab.org/protocols) with modifications. After 5 days of infection with lent-shRNA virus, neurons were fixed in 4% formaldehyde for 15 min and permeabilized with 0.8% Triton-X 100 in 1 × SSC for 30 min at room temperature. Digoxigenin-labelled cRNA corresponding to the sense or antisense strand of *Rab3a* and *Aplp1* were generated using PCR fragments containing 5′-end of T7 (5′- CCAAGCTTCTAATACGACTCACTATAGGGAGA -3′) or Sp6 (5′- GATTTAGGTGACACTATAGAAG -3′) RNA polymerase promoter sequences. Cells were hybridized overnight at 50 °C. Digoxigenin-labelled cRNA was detected with a sheep anti-digoxigenin antibody (1:200, Roche) for 1 h at 37 °C followed by Alexa 488-donkey anti-sheep antibody (1:300) for 1 h at room temperature.

### Electrophysiology

Whole-cell patch clamp recordings were made from neurons under continuous perfusion of a medium containing (in mM) 119 NaCl, 2.5 KCl, 2.8 CaCl_2_, 2 MgCl_2_, 26 NaHCO_3_, 1 NaH_2_PO_4_, 11 glucose, 0.1 picrotoxin (Sigma) and 0.0005 tetrodotoxin (Tocris Bioscience) with a osmolarity of 290 mOsm. It was gassed with 5% CO_2_/95% O_2_ to maintain oxygenation and a pH of 7.4. The solution within the patch pipette consisted of (in mM): 115 caesium methanesulfonate, 20 CsCl, 10 HEPES, 2. 5 MgCl_2_, 4 ATP disodium salt, 0.4 GTP trisodium salt, 10 sodium phosphocreatine and 0.6 EGTA, at pH 7.25. mEPSCs were recorded at −65 mV membrane potential using an Axopatch 200B amplifier (Axon Instruments, Union City, CA), low-pass filtered at 2 kHz and digitized at 5 kHz with a Digidata 1,320 A (Axon Instruments). mEPSCs were analysed using the pClamp 9 (Axon Instruments). All the detected events were re-examined and accepted or rejected on the basis of visual examination. Cells were recorded from for roughly 5 min to obtain at least 100 events per cell. Data obtained from the indicated number (*n*) of cells were expressed as the mean±s.e.m. and analysed using Student's *t*-test. Recording and data analyses were performed without prior knowledge of the treatment history of the cultures.

### Statistical analyses

All values are the mean and s.e.m. of the number of biological replicates noted in the Figure legends. Student's *t*-test was used for analysis of data from experiments that involved only two conditions (control and experimental treatment). For experiments involving more than two conditions, one-way analysis of variance was performed followed by Dunnett's *post hoc* analysis, unless otherwise indicated in the figure legends.

## Additional information

**Accession codes:** Microarray data (NCBI GEO accession numbers: GSE72887 and GSM1874108–GSM187414).

**How to cite this article:** Zhang, P. *et al*. Novel RNA- and FMRP-binding protein TRF2-S regulates axonal mRNA transport and presynaptic plasticity. *Nat. Commun.* 6:8888 doi: 10.1038/ncomms9888 (2015).

## Supplementary Material

Supplementary Information Supplementary Figures 1-14 and Supplementary Tables 1-3.
