# Supplementary material for: Novel RNA- and FMRP-binding protein TRF2-S regulates axonal mRNA transport and presynaptic plasticity
Source: Nat Commun. 2015 Nov 20;6:8888. doi: 10.1038/ncomms9888 (PMC4673492; doi:10.1038/ncomms9888)
Supplement: Supplementary Information — Supplementary Figures 1-14 and Supplementary Tables 1-3. [file ncomms9888-s1.pdf]

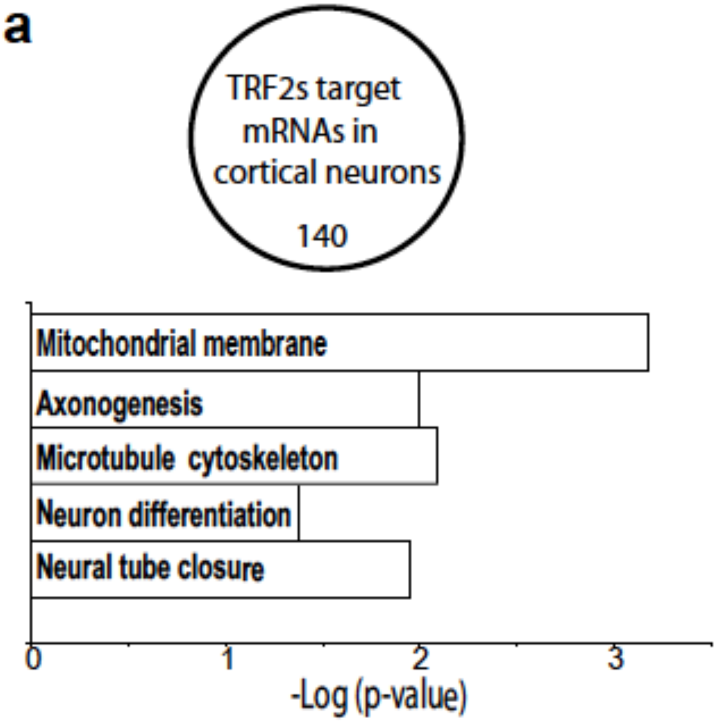


**b**

Putative TRF2-S target axonal mRNAs

| | |
|---|---|
| RAB3A | RPL18A |
| APLP1 | RPL13A |
| RAB2 | LOC499793 |
| SYPL | Rpl7 |
| GDI1 | NNAT |
| GNG10 | MAP1B |
| NREP | Map1lc3a |
| Akt1 | MAPRE1 |
| Brsk1 | MTAP2 |
| PPP2R5B | Nckap1 |
| YY1 | ATP5G3 |
| HMGB2 | SLC25A11 |
| TXN1 | COX7A2L |
| CIC | BAX |
| Cox7b | Ndufb2 |
| TOMM22 | NDUFB3 |

**Supplementary Figure 1 |** (**a**) Profiling of TRF2-S-associated transcriptomes in cortical neurons. Ribonucleoprotein (RNP) immunoprecipitation (RIP)-assay and subsequent microarray analysis identified 140 putative TRF2-S bound transcripts in cultured rat cerebral cortical neurons. A bar graph shows the top gene ontology (GO) terms that are enriched in TRF2-S targets. The GO terms are ranked by -log *P* values. (**b**) A list of putative TRF2-S target axonal mRNAs.

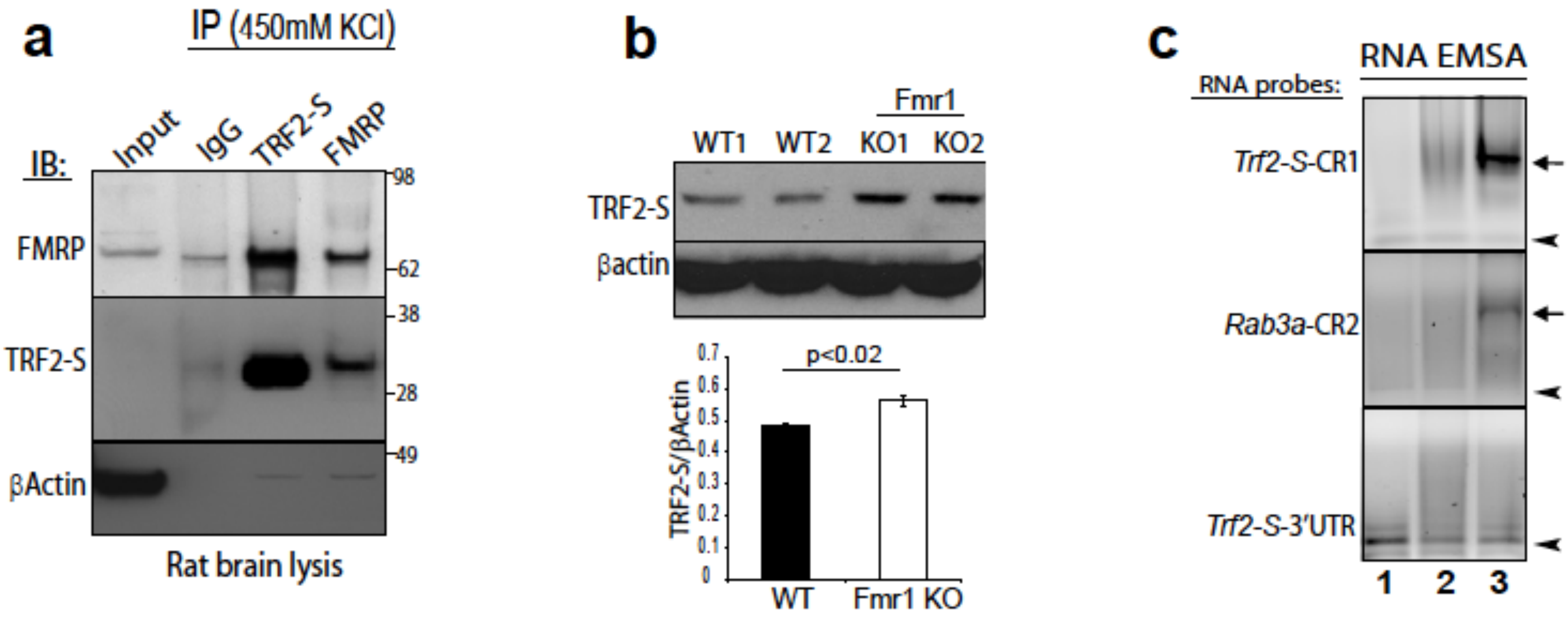


**Supplementary Figure 2 |** (**a**) CoIP of TRF2-S and FMRP with brain cytoplasmic extracts using 450 mM KCl IP buffer. The immunoblots demonstrate that endogenous TRF2-S and FMRP interact with each other under the high salt IP condition. (**b**) WT and *Fmr1* KO brain lysates were subjected to immunoblotting using FMRP and TRF2-S antibodies. Relative protein loading was assessed by bActin. Bottom, densitometry analysis of TRF2-S normalized to bActin. n=3 per group. All values are mean$\pm$SD. P values are based on Student's *t*-test. (**c**) RNA mobility shift assay showing that the binding of FMRP with TRF2-S inhibited the formation of TRF2-S-mRNA complex. The lysate from HEK293 cells expressing HA-TRF2-S was mixed either with or without recombinant GST-FMRP protein prior to HA-CoIP. The purified protein complexes were than incubated with TRF2-S target RNA fragments *Trf2*-S-CR1 (upper), *Rab3*a-CR2 (middle) and a non-target RNA fragment *Trf2*-S-3'UTR (lower) for RNA-EMSA. Lane 1, GST-FMRP only; Lane 2, GST-FMRP plus HA-TRF2-S; lane 3, HA-TRF2-S only. Arrow and arrowhead point to bound and unbound mRNA fragments, respectively.

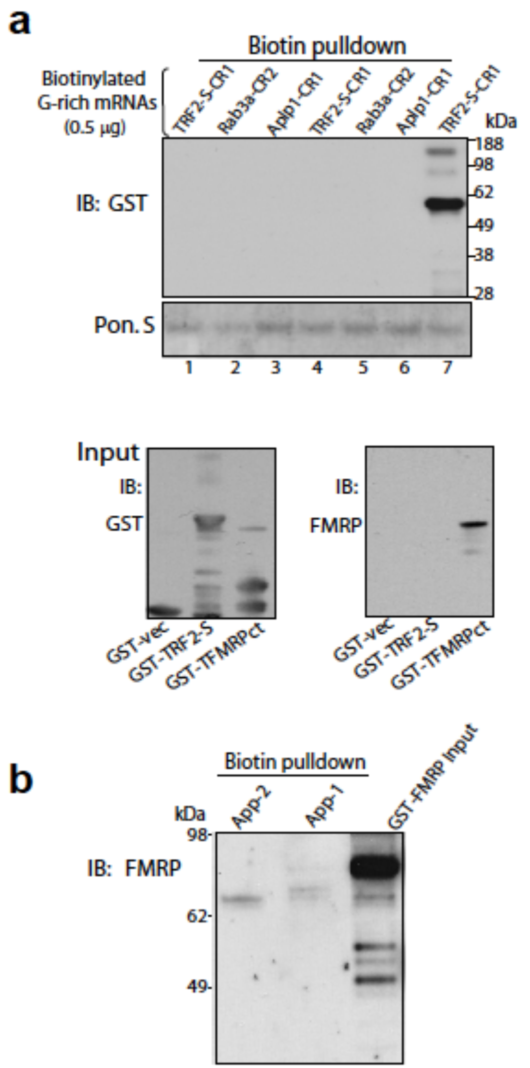


**Supplementary Figure 3 |** (**a**) Top, biotin pulldown and immunoblotting showing that neither recombinant GST empty vector (lane 1-3) nor GST-FMRPct (lane 4-6) that contains a truncated mouse FMRP with RNA binding RGG box were capable of binding to TRF2-S target mRNAs. GST-TRF2-S (lane7) served as positive control. Bottom, input of GST tagged recombinant proteins. (**b**) Biotin pulldown and immunoblotting showing that recombinant GST-FMRP was capable of binding to biotinylated *App1* and *App2*, two *App* fragments known as FMRP targets[41,42].

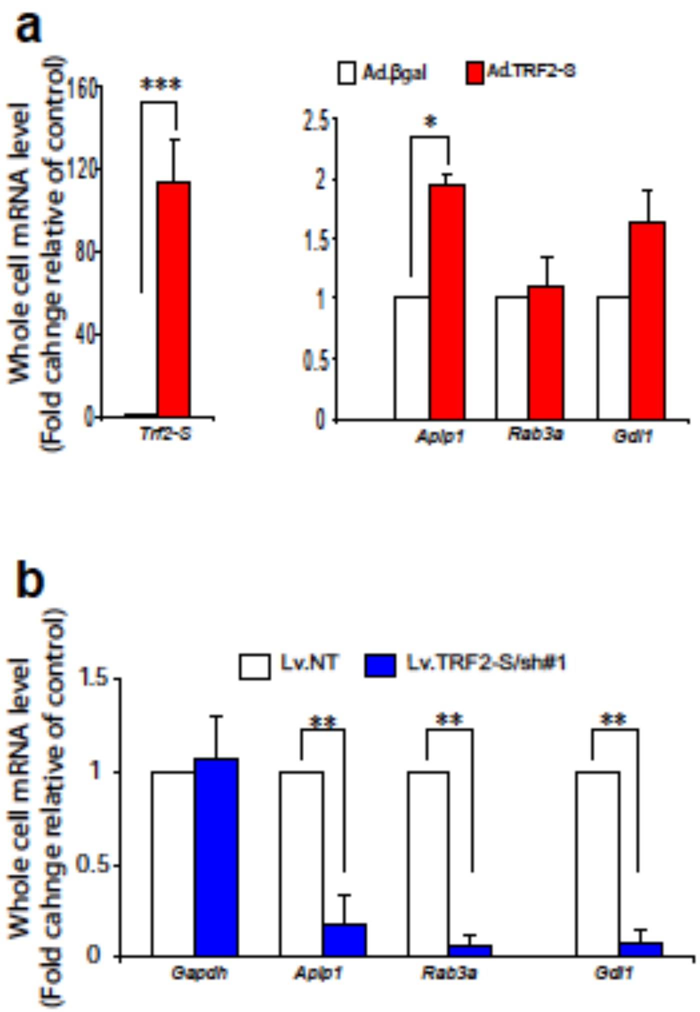


**Supplementary Figure 4 |** (**a-b**) On culture day 6 cortical neurons were infected for 4 days with adenovirus bearing βGal and TRF2-S (a), and lentivirus bearing shRNA#1 (865) for TRF2-S silencing and non-target control (b). The whole cell extracts were subjected for RT-qPCR analysis of TRF2-S target mRNAs. The values are the fold change normalized to*18S rRNA*. *Gapdh* is a non-target mRNA of TRF2-S. n=3. All values are mean±SD. P values are based on Student's *t*-test.

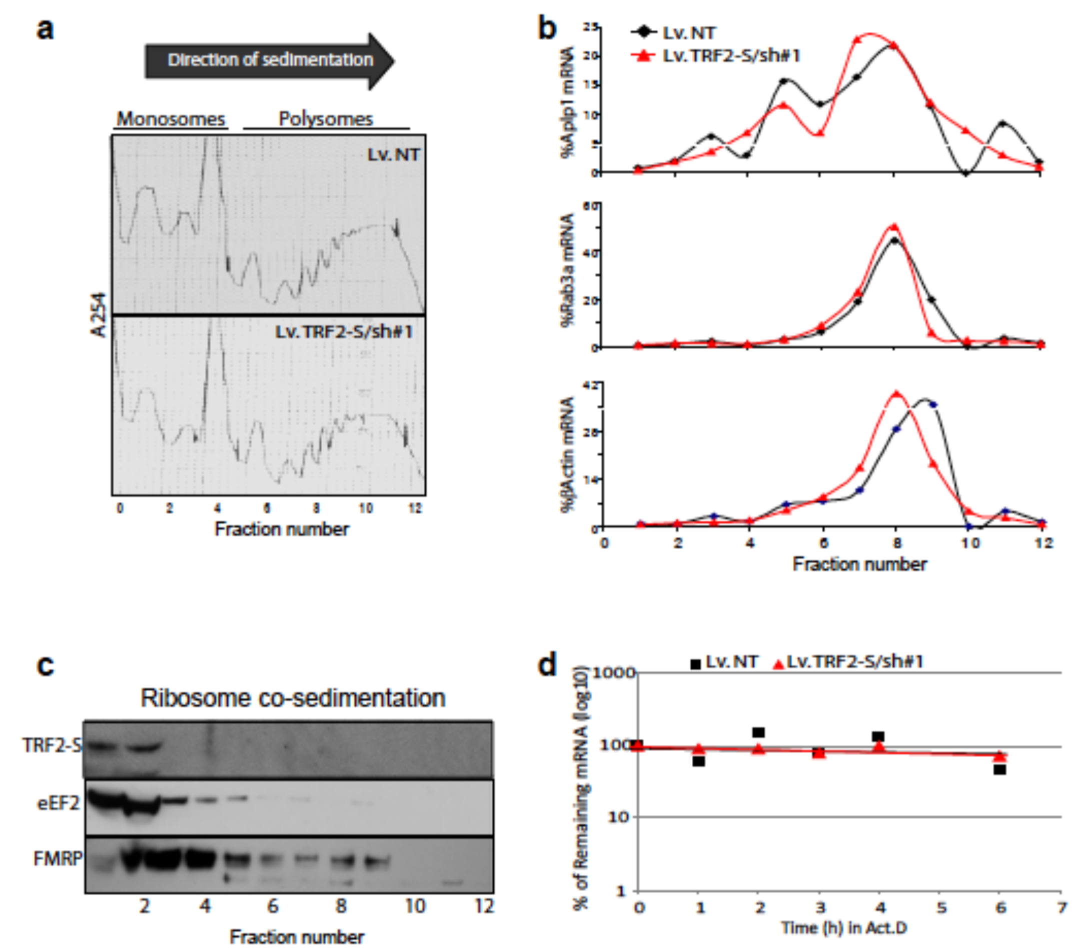


**Supplementary Figure 5 |** TRF2-S exhibits a monosomal sedimentation profile, and has little or no global effects on translations of its target mRNA. (**a**) On culture day 6 cortical neurons were infected for 4 days with lentivirus carrying non-target (NT) scramble shRNA and TRF2s targeting shRNA#1. Whole cell lysates were fractionated through linear sucrose gradients (15-60%). The arrow indicates the direction of sedimentation from monosomes to polysomes. (**b**) The relative distributions (percentages) of *Aplp1*, *Rab3a* and *Actb* mRNAs were quantified by RT-qPCR analysis of RNA in each of 12 gradient fractions. (**c**) TRF2-S, FMRP and eEF2 protein distributions in each of 12 gradient fractions from the cortical neuron lysate (9 d in culture) were analyzed by immunoblotting. (d) On culture day 6 cortical neurons were subjected to lentiviral mediated TRF2-S silencing as described in a. Four days later neurons were treated with actinomycin D. The RNAs were collected at the indicated times. The half-life of *Aplp1*mRNA was analyzed by RT-qPCR upon normalization to *Gapdh*, defined as the time needed to reach 50% of its original abundance at time 0 h. The linear-fit lines are for Lv. NT-control (black) and Lv. TRF2-S/sh#1(red), respectively. The graph represents two independent experiments.

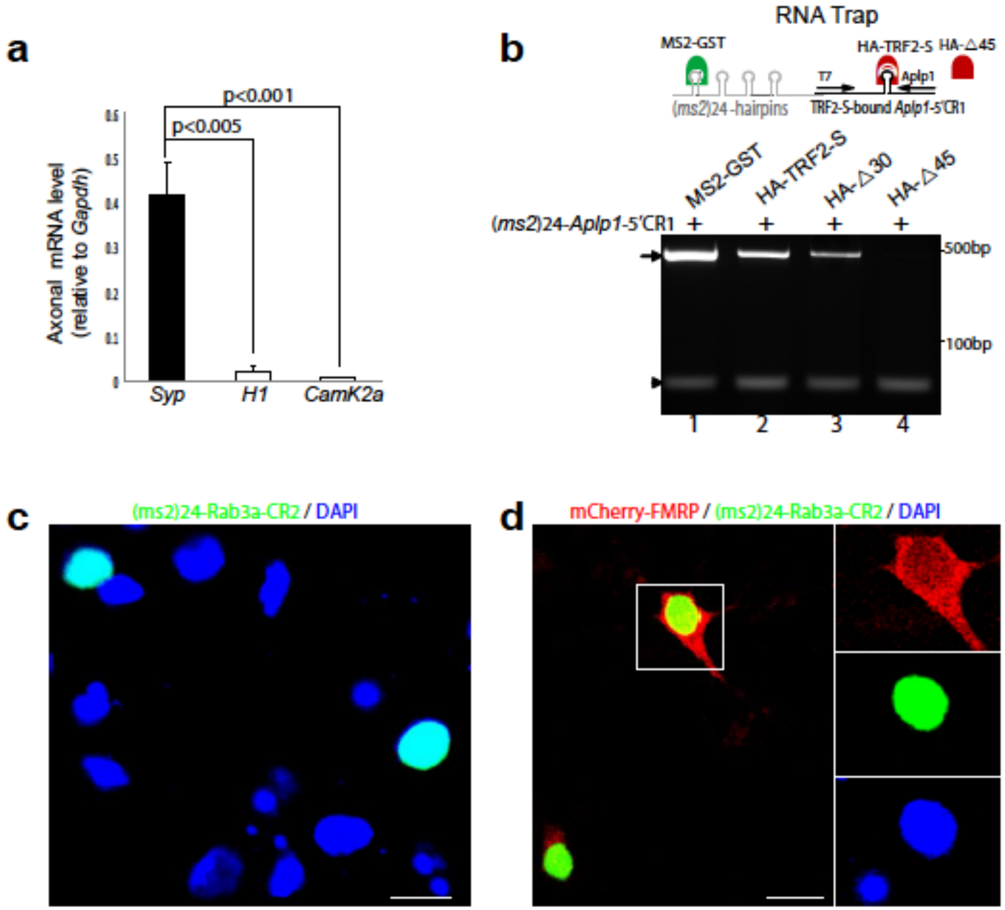


**Supplementary Figure 6 |** (**a**) RT-qPCR analysis of axonal mRNAs harvested from the high-throughput microfluidic system after 9 d cortical neuron cultures. Upon the normalization with *Gapdh*, the axonal mRNA *synaptophysin* (*Syp*) was readily detectable, whereas the levels of dendritic mRNA *calcium/calmodulin-dependent protein kinase II* (*CamK2a*) and nuclear mRNA *Histone1*(*H1*) were barely measurable (average Ct values are larger than 33.6 and 32.3, respectively). n=3. All values are mean±SD. P values are based on Student's *t*-test. (**b**) Top, schematic of RNA trap assay and necessities of TRF2-S GAR domain and MS2-GST for precipitation of a (*ms2*)24 fused TRF2-S target RNA chimera. Bottom, HEK293 cells were co-transfected with the plasmid expressing (*ms2*)24 tagged *Aplp1*-5'CR1 RNA chimera, accompanied with either MS2-GST or HA-TRF2-S variants. The existence of the exogenous RNA chimera in each GST- or HA-precipitates was assessed by RT-PCR using a primer set (T7 and Aplp1). An arrow points to PCR products; an arrowhead points to primer dimmers. (**c-d**) DIV7 cortical neurons were co-transfected with MS2-YFP-NLS and (*ms2*)24-*Rab3a*-CR2 without (**c**) or with mCherry-FMRP (**d**). Overexpression of mCherry-FMRP failed to translocate YFP labeled RNP from the nucleus. The images are representative from 50 transfected neurons per group. Scale bar=20μm.

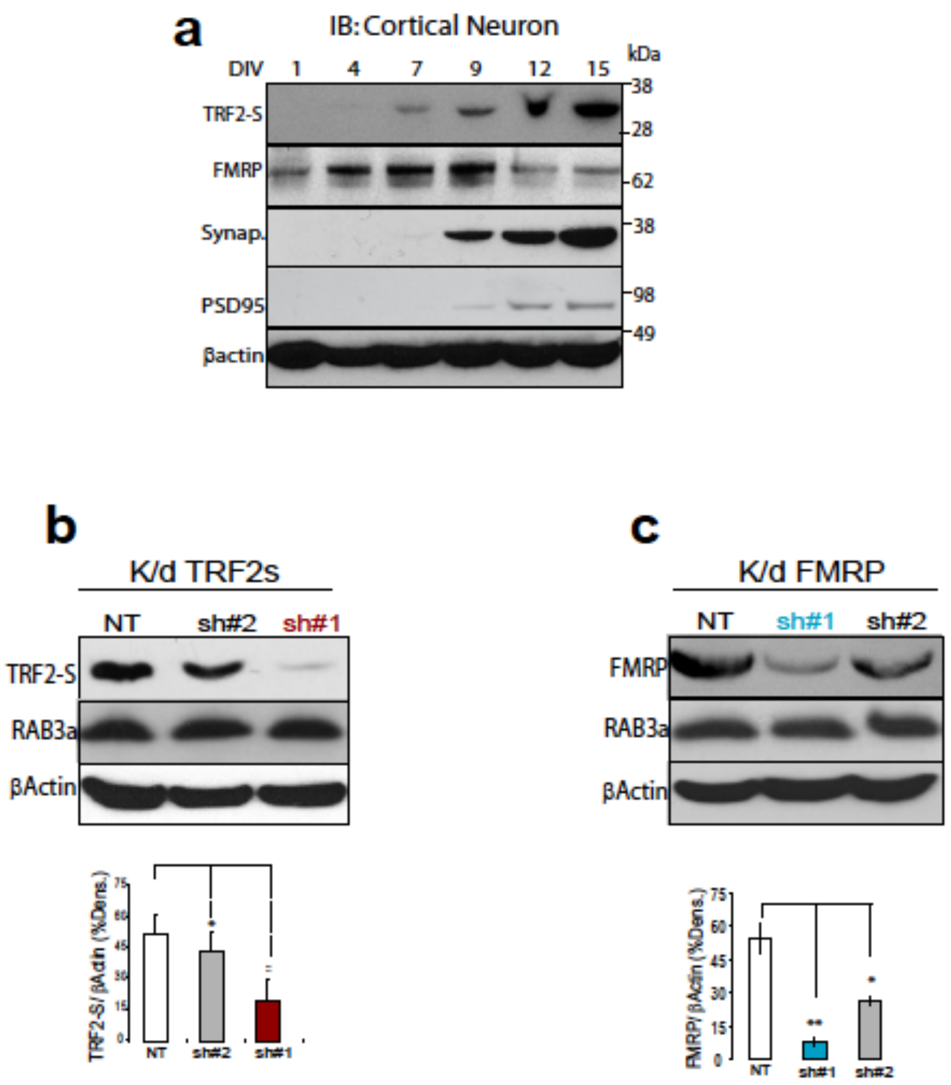


**Supplementary Figure 7 |** (**a**) Immunoblots showing the expression patterns of TRF2-S and FMRP during the course of neuronal maturation. Synaptophysin (Synap) and PSD95 are pre-and post-synaptic markers, respectively. (**b-c**) Neurons (6 days in culture) were infected for 4 days with lentivirus carrying shRNAs for the silencing of rat TRF2-S (shRNA#1, 865 and shRNA#2, 774) in **b**; for the silencing of rat FMRP (shRNA#1, 2624 and shRNA#2, 2623) in **c**. A non-target (NT) scramble shRNA was used as controls. Cell lysates were then subjected to immunoblot analysis of endogenous levels of Rab3a, along with TRF2-S (**b**) and FMRP (**c**). Densitometry analysis showed as bar graphs at bottom of b and c. n=3; *p<0.05 and **p<0.005. All values are mean±SD. P values are based on Student's *t*-test.

Full scan for western blot in Figure 1 c

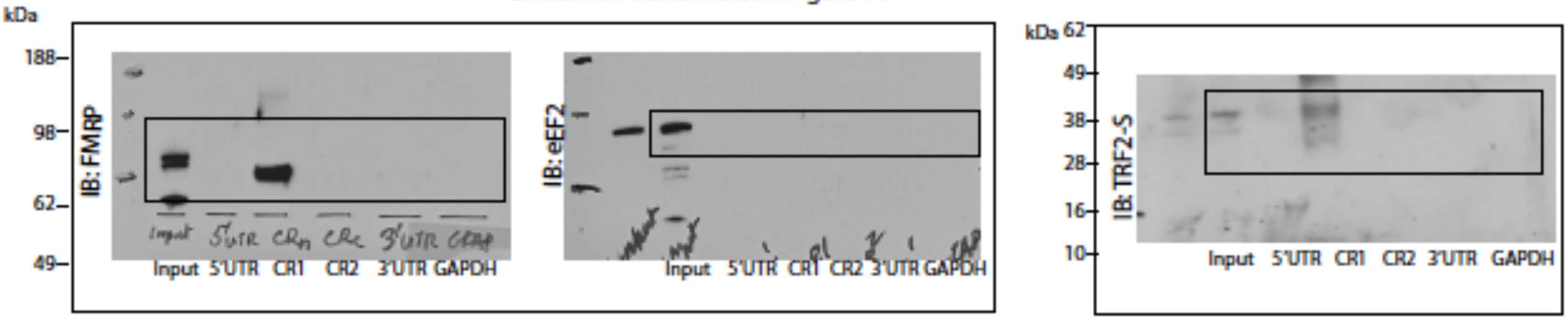


Full scan for blots in Figure 1 d

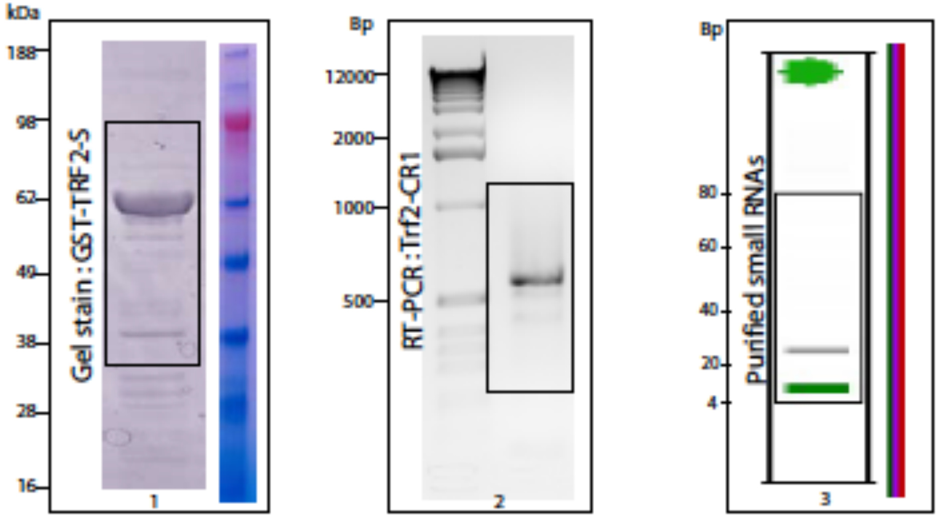


Full scan for blots in Figure 1 e

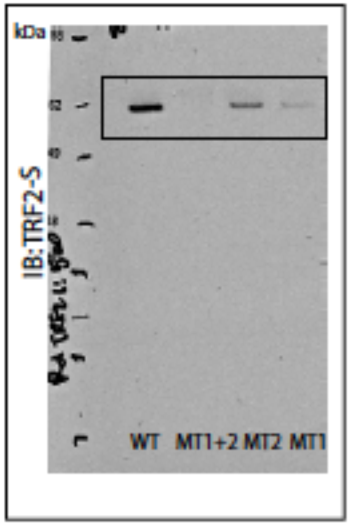


**Supplementary Figure 8.** Full blots for Figures 1c, d and e.

Full scan for western blots in Fig. 2a

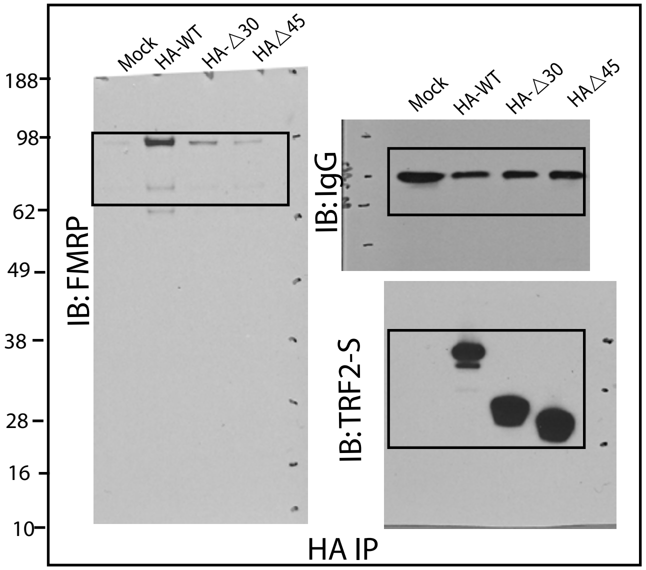


Full scan for western blots in Fig. 2b

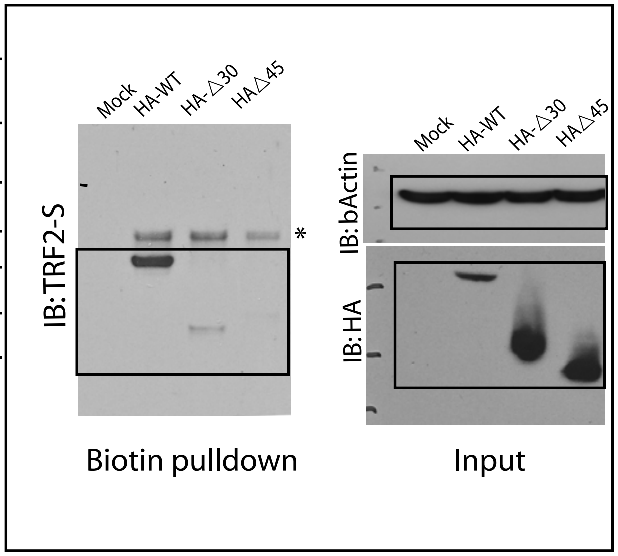


* Endogenous TRF2 in 293 cells

Full scan for western blots in Fig. 2c

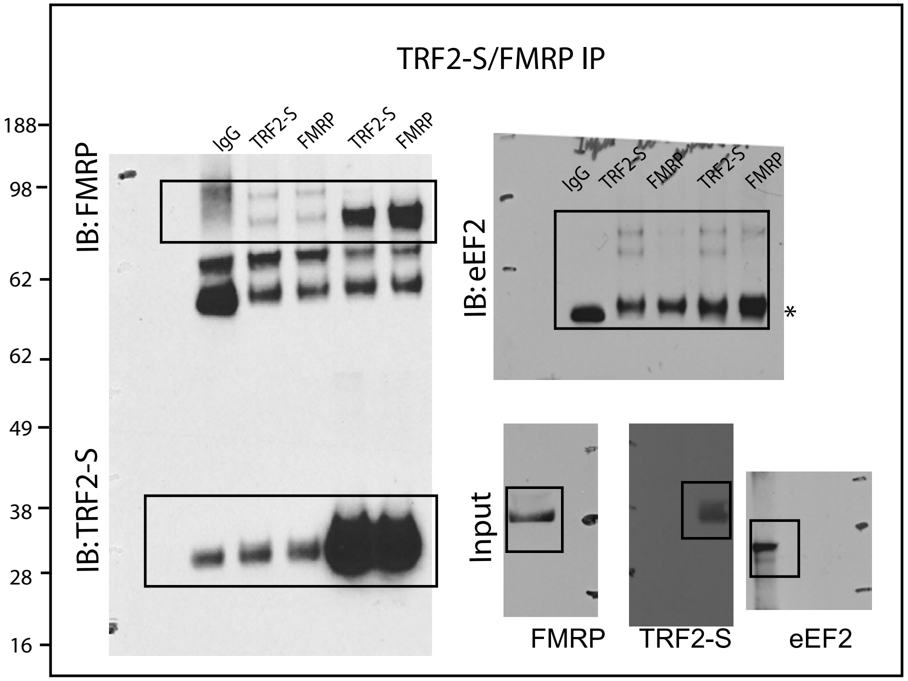


* IgG

Full scan for western blots in Fig. 2d

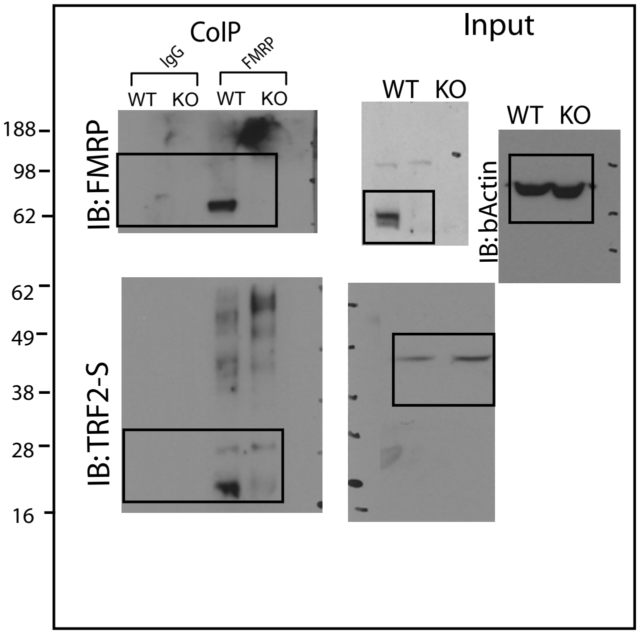

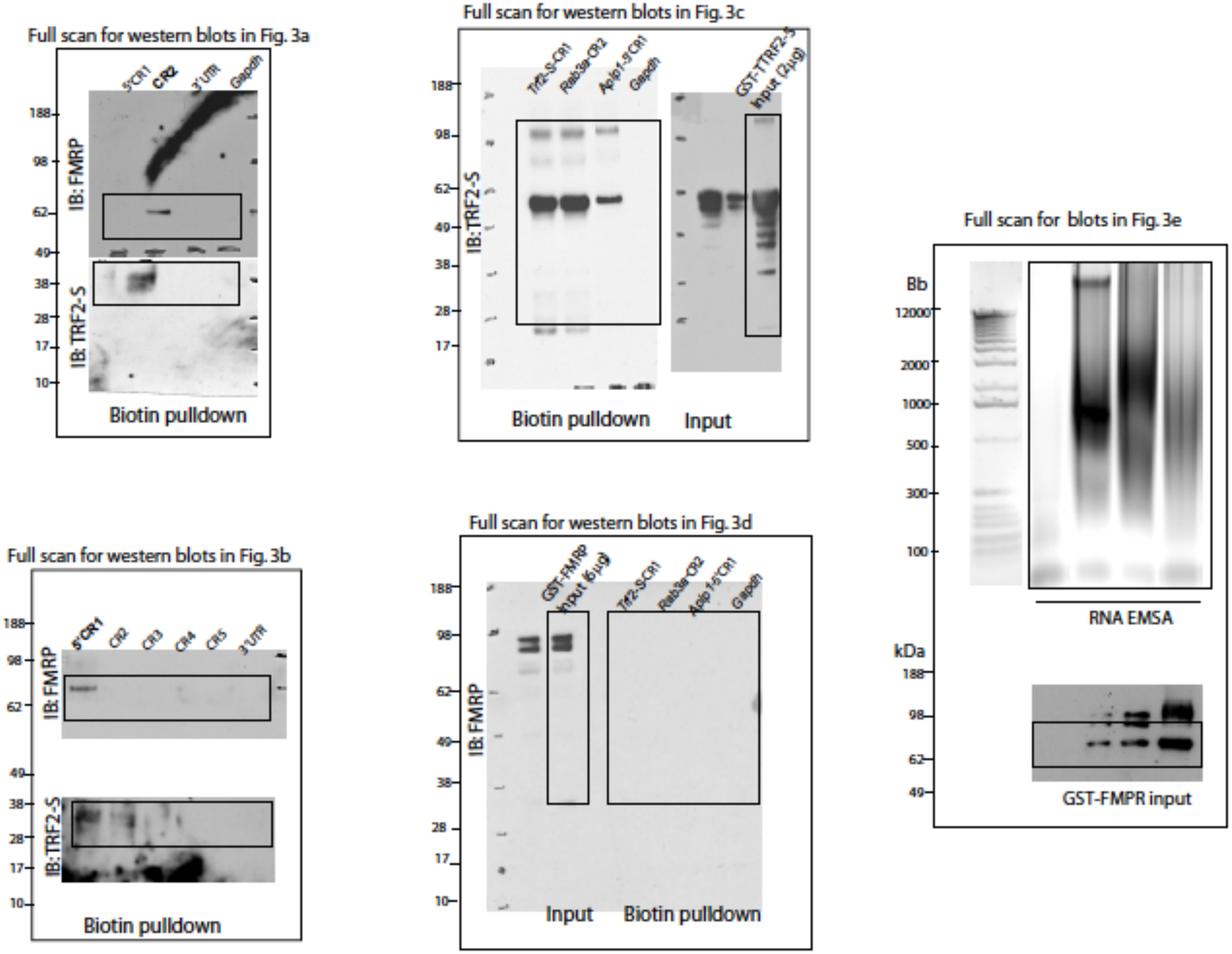


**Supplementary Figure 10.** Full blots for Figures 3 a, b c, d and e.

Supplementary Figure 11

Full scan for western blots in Supplementary Fig. 2a

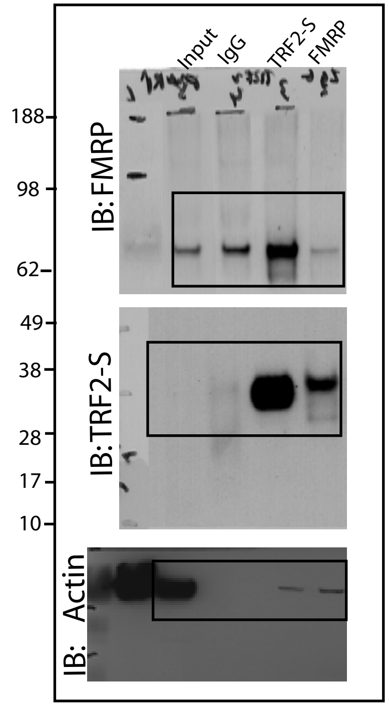


Full scan for western blots in Supplementary Fig. 2b

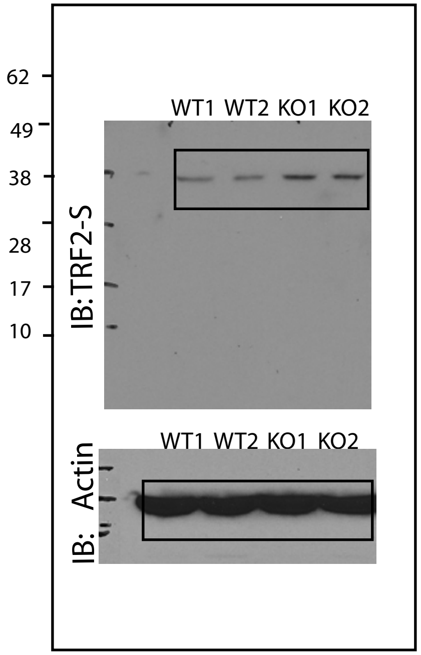


Full scan for western blots in Supplementary Fig. 2c

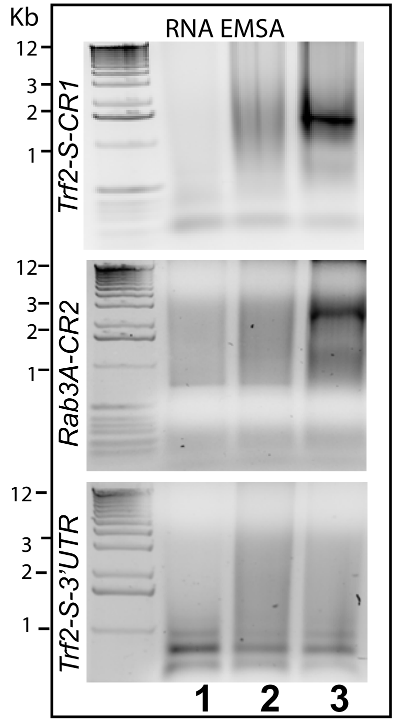

Full scan for western blots in Supplementary Fig. 3a

Full scan for western blots in Supplementary Fig. 3b

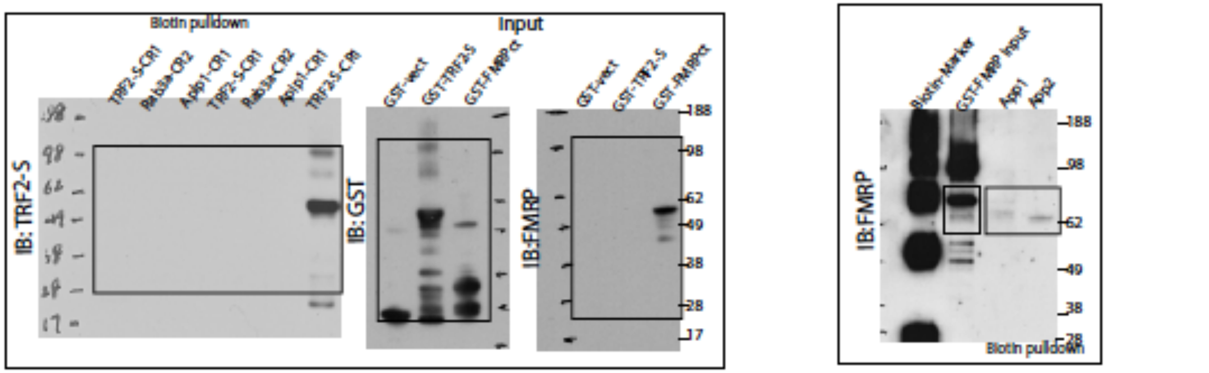


**Supplementary Figure 12.** Full blots for Supplementary Figures 3 a and b.

Full scan for western blots in Supplementary Fig. 5c

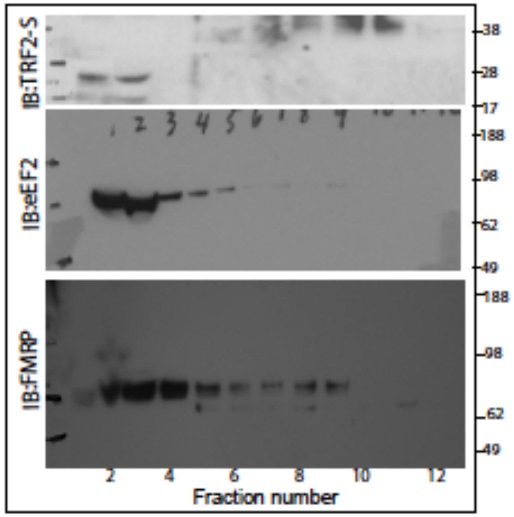


**Supplementary Figure 13.** Full blot for Supplementary Figure 5c.

Full scan for western blots in Supplementary Fig. 7a

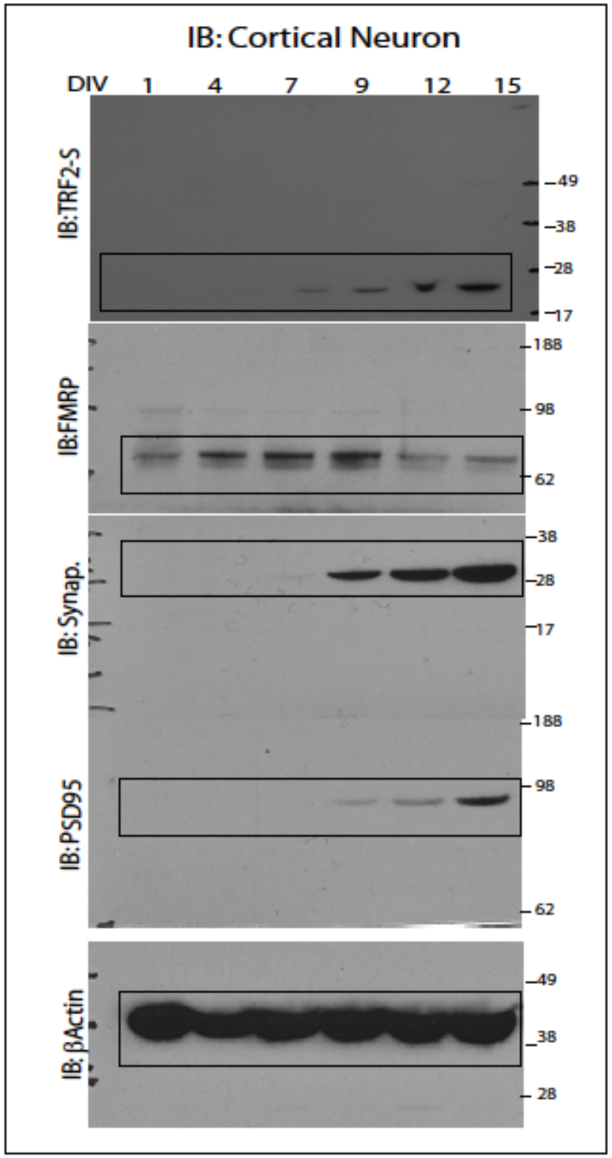


Full scan for western blots in Supplementary Fig. 7b

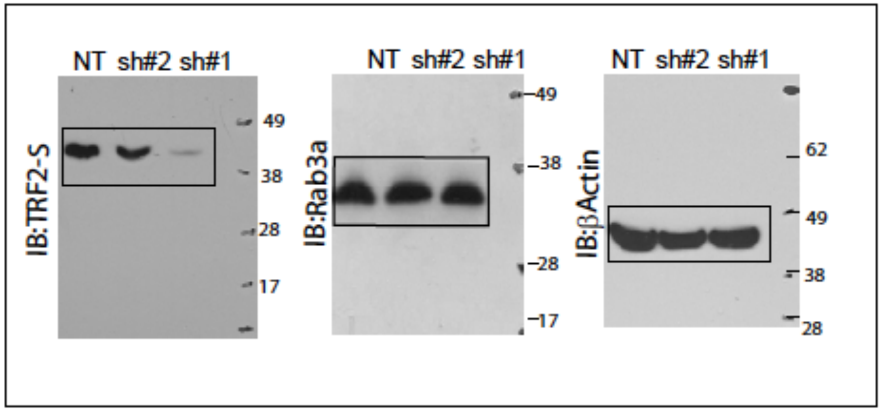


Full scan for western blots in Supplementary Fig. 7c

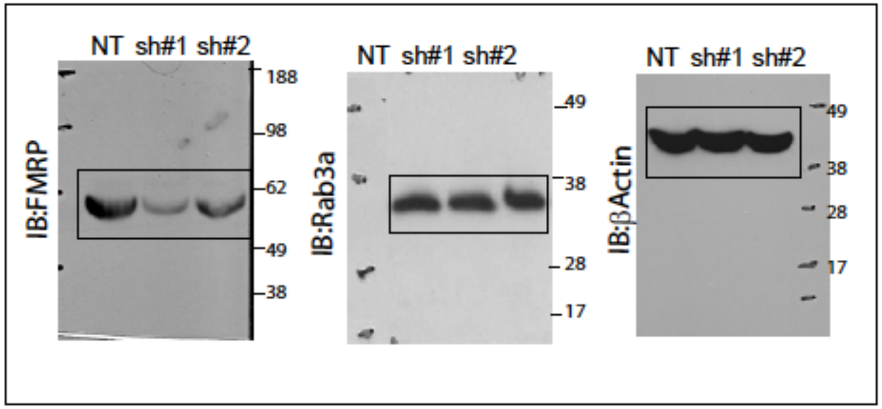


**Supplementary Figure 14.** Full blots for Supplementary Figures 7a, b and c.

Supplemental table1. **Global analysis of TRF2-S bound neural mRNAs**

| Gene Symb | group 1 Z ratio | group 2 Z ratio | RefSeq ID | GI | Description |
|---|---|---|---|---|---|
| MLPH | 14.07 | 8.5 | NM_001012135.1 | 58865839 | melanophilin (Mlph) |
| Akt1 | 9.67 | 5.85 | NM_033230 | 402692490 | thymoma viral proto-oncogene 1 (Akt1) |
| NNAT | 9.12 | 9.85 | NM_181687 | 399498546 | neuronatin (Nnat) |
| Gcfc2 | 8.79 | 5.37 | NM_001134554 | 197386558 | GC-rich sequence DNA-binding factor 2 (Gcfc2) (RGD1304792_predicted) |
| LOC501280 | 8.59 | 7.88 | XR_007805 | 109486608 | similar to myosin regulatory light chain-like (LOC501280) |
| EIF4EBP2 | 8.41 | 6.8 | NM_001033069.1 | 74271858 | eukaryotic translation initiation factor 4E binding protein 2 (Eif4ebp2) |
| MLLT11 | 8.4 | 7.71 | NM_001013912.1 | 62078554 | myeloid/lymphoid or mixed-lineage leukemia (trithorax homolog |
| LOC500095 | 8.33 | 4.95 | XM_575446 | 62647113 | similar to T-cell receptor beta chain V region 86T1 precursor (LOC500095) |
| TEKT1 | 7.96 | 3.54 | NM_053508.2 | 51036667 | tektin 1 (Tekt1) |
| EPN1 | 7.88 | 8.21 | NM_057136.1 | 16923989 | Epsin 1 (Epn1) |
| MAPK3 | 7.41 | 1.95 | NM_017347 | 68537200 | mitogen activated protein kinase 3 (Mapk3) |
| MLF2 | 7.03 | 9.95 | NM_001107889 | 157823872 | myeloid leukemia factor 2 (predicted) (Mlf2_predicted) |
| SLC39A2 | 6.94 | 4.33 | NM_001107260 | 157822388 | solute carrier family 39 (zinc transporter) |
| R3hdm2 | 6.54 | 3.1 | NM_001130557 | 194474025 | R3H domain containing 2 |
| PFN1 | 6.306 | 7.966 | NM_022511.2 | 42476143 | profilin 1 |
| RGD1564087 | 6.1 | 2.47 | XR_009360.1 | 109467794 | similar to 14-3-3 protein tau (14-3-3 protein theta) (predicted) |
| RGD1559566 | 5.99 | 4.83 | XM_001056263.1 | 293350680 | similar to 60S ribosomal protein L9 (predicted) (RGD1559566_predicted) |
| ATP5G3 | 5.997 | 7.804 | NM_053756 | 16758591 | ATP synthase, H+ transporting, mitochondrial Fo complex, subunit C3 (subunit 9) |
| CIC | 5.98 | 5.89 | NM_001107490.3 | 215272395 | capicua homolog (Drosophila) (predicted) (Cic_predicted) |
| Brsk1 | 5.93 | 7.05 | NM_001127337 | 187960159 | BR serine/threonine kinase 1 |
| CAMK2N2 | 5.63 | 4.92 | NM_021678.1 | 11067400 | calcium/calmodulin-dependent protein kinase II inhibitor 2 (Camk2n2) |
| RGMA | 5.51 | 4.83 | XM_218791.3 | 157819830 | RGM domain family |
| Ccdc125 | 5.25 | 3.49 | NM_001134761 | 197927213 | CCDC125 coiled-coil domain containing 125 |
| GNG10 | 5.21 | 7.74 | NM_053660.1 | 399154111 | guanine nucleotide binding protein (G protein) |
| P11 protein | 5.05 | 2.51 | XM_579635.1 | 62659845 | similar to P11 protein (LOC497693) |
| Tmem189 | 5.02 | 2.58 | NM_001113752 | 166091516 | transmembrane protein 189 |
| LOC365025 | 5.15 | 6.63 | XM_577671.1 | 62665500 | similar to alpha tubulin subunit (LOC365025) |
| SNURF | 5.12 | 10.02 | NM_130738.1 | 397787561 | SNRPN upstream reading frame (Snurf) |
| LOC300760 | 5.03 | 3.57 | XM_236308.3 | 62653977 | similar to H3 histone |
| Yaf2 | 5.063 | 5.468 | XM_001073895.1 | 199559858 | similar to YY1-associated factor 2 (LOC690262) |
| LOC500344 | 4.9 | 2.49 | XM_575696.1 | 62648245 | LOC500344 (LOC500344) |
| MRPS2 | 4.88 | 3.8 | NM_001108576 | 157819458 | mitochondrial ribosomal protein S2 |

| | | | | | |
|---|---|---|---|---|---|
| TOMM22 | 4.63 | 4.9 | NM_212514.2 | 71534292 | translocase of outer mitochondrial membrane 22 homolog (yeast) (Tomm22) |
| Arglu1 | 4.63 | 4.5 | NM_001024972.1 | 68163363 | arginine and glutamate rich 1 () |
| TMEM158 | 4.61 | 6.33 | NM_057212.1 | 17933495 | transmembrane protein 158 (Tmem158) |
| LY6H | 4.44 | 8.82 | NM_001134839.1 | 198386344 | lymphocyte antigen 6 complex |
| LENG4 | 4.31 | 3.55 | NM_001134978 | 201066394 | leukocyte receptor cluster (LRC) member 4 |
| AKT1S | 4.25 | 3.54 | NM_001106259 | 408772021 | AKT1 substrate 1 (proline-rich) |
| Zgc:56193 | 4.23 | 5.55 | XR_008163.1 | 109477420 | similar to Zgc:56193 (predicted) (RGD1562162_predicted) |
| WBP2 | 4.21 | 3.32 | NM_138975.1 | 20376817 | WW domain binding protein 2 (Wbp2) |
| CYP46A1 | 4.11 | 3.77 | NM_001108723. | 157817886 | cytochrome P450 |
| Btbd2 | 4.11 | 5.43 | XM_001076264.1 | 392341333 | similar to BTB (PO)Z domain containing 2 (predicted) (RGD1566094_predicted) |
| CSDC2 | 4.08 | 3.7 | XM_001077363.1 | 293348836 | cold shock domain containing C2 |
| RPL13A | 4.05 | 8.07 | NM_173340.1 | 27545437 | ribosomal protein L13A (Rpl13a) |
| RGD1564980 | 4.02 | 4.16 | XM_575373.2 | 293358409 | similar to 60S ribosomal protein L29 (P23) (predicted) (RGD1564980_predicted) |
| Prrc2a | 3.99 | 3.82 | NM_212462.2 | 260166645 | proline-rich coiled-coil 2A (Prrc2a) |
| PPP2R5B | 3.93 | 7.09 | NM_181379.2 | 60593006 | protein phosphatase 2 |
| LOC681314 | 3.91 | 5.43 | XM_001061020.1 | 109489050 | similar to BAX protein |
| TXN1 | 3.87 | 3.53 | NM_053800.2 | 42476285 | thioredoxin 1 (Txn1) |
| MFAP2 | 3.81 | 3.89 | NM_001107989 | 157818962 | microfibrillar-associated protein 2 (predicted) (Mfap2_predicted) |
| HMG-2 | 3.8 | 3.27 | XM_573272.1 | 392351998 | similar to High mobility group protein 2 (HMG-2) (LOC498072) |
| Hmgb2 | 3.8 | 2.76 | NM_017187 | 158711705 | high mobility group box 2 (Hmgb2) |
| LOC364253 | 3.76 | 4.27 | XM_344296.1 | 34869079 | similar to RIKEN cDNA 1110003E01 (LOC364253) |
| ADCK2 | 3.72 | 1.75 | NM_001107855 | 157822890 | aarF domain containing kinase 2 (predicted) (Adck2_predicted) |
| RPS7 | 3.71 | 2.15 | XM_213053.2 | 62650073 | ribosomal protein S7 (Rps7) |
| Ppp1r18 | 3.69 | 4.46 | NM_001126287.2 | 261399901 | PPP1R18 protein phosphatase 1, regulatory subunit 18 |
| B3GAT1 | 3.66 | 4.35 | NM_054003.1 | 16758917 | beta-1,3-glucuronyltransferase 1 |
| RGD1565149 | 3.66 | 4.35 | XM_001077968.1 | 293343422 | similar to chromosome 16 open reading frame 7 (predicted) (RGD1565149_predicted) |
| Fam57b | 3.62 | 7.49 | NM_001106296.2 | 182888556 | family with sequence similarity 57, member B |
| LOC306766 | 3.61 | 3.23 | NM_001014007.1 | 62078742 | hypothetical LOC306766 (LOC306766) |
| RGD1311783 | 3.59 | 4.89 | XM_001059424.1 | 197386190 | similar to RIKEN cDNA 2010012O05 (predicted) (RGD1311783_predicted) |
| Ptbp2 | 3.52 | 1.87 | NM_001005555.1 | 53850637 | polypyrimidine tract binding protein 2 (Ptbp2) |
| NME2 | 3.51 | 4.75 | NM_031833.2 | 55926144 | NME/NM23 nucleoside diphosphate kinase 2 |
| Agap3 | 3.47 | 5.59 | NM_001108616 | 157824114 | ArfGAP with GTPase domain, ankyrin repeat and PH domain 3 |
| Rab2a | 3.468 | 1.64 | NM_031718.1 | 13929005 | member RAS oncogene family (Rab2a) |
| MGST3 | 3.46 | 4.62 | XM_001076104.1 | 109498992 | microsomal glutathione S-transferase 3 (predicted) (Mgst3_predicted) |
| Gprin1 | 3.45 | 3.49 | XM_001070221.1 | 293342617 | G protein-regulated inducer of neurite outgrowth 1 (predicted) (Gprin1_predicted) |
| LOC500721 | 3.45 | 5.65 | XM_580114.1 | 62651251 | LOC500721 (LOC500721) |
| Gstp1 | 3.45 | 4.18 | NM_012577.1 | 169646324 | glutathione-S-transferase |
| Nckap1 | 3.41 | 2.03 | XM_230038.4 | 158138548 | NCK-associated protein 1 (Nckap1) |
| RRS1 | 3.394 | 6.446 | NM_001079699.1 | 119226199 | RRS1 ribosome biogenesis regulator homolog (S.cerevisiae) |
| NREP | 3.38 | 7.02 | NM_178096.2 | 31342528 | neuronal regeneration related protein (Nrep) |
| RGD1561956 | 3.37 | 3.52 | XM_344287.1 | 34869055 | similar to RIKEN cDNA 1110003E01 (predicted) (RGD1561956_predicted) |

| | | | | | |
|---|---|---|---|---|---|
| GDI1 | 3.35 | 5.95 | NM_017088.2 | 71534275 | guanosine diphosphate dissociation inhibitor 1 (Gdi1) |
| RGD1564450 | 3.35 | 3.26 | XM_001078024.1 | 157822410 | RGD1564450 (predicted) (RGD1564450_predicted) |
| RGD1560015 | 3.28 | 4.26 | XM_574311.2 | 392343688 | trans-2,3-enoyl-CoA reductase-like |
| Npm3 | 3.26 | 3.95 | XM_001058548.1 | 392338228 | nucleophosmin/nucleoplasmin, 3 |
| Zbtb45 | 3.26 | 3.37 | NM_001107478 | 157819040 | zinc finger and BTB domain containing 45 |
| SPC25 | 3.26 | 2.23 | NM_001009654.1 | 57527006 | SPC25, NDC80 kinetochore complex component |
| LOC311629 | 3.21 | 3.81 | XM_230844.3 | 62646356 | similar to sorting nexin 21 isoform a; sorting nexin L; sorting nexin 21 (predicted) |
| PPFIA3 | 3.21 | 3.91 | NM_001270985 | 401709972 | protein tyrosine phosphatase, receptor type |
| Cox7b | 3.2 | 2.29 | NM_182819.2 | 65301489 | cytochrome c oxidase subunit VIIb |
| Gtf2h5 | 3.17 | 1.82 | NM_001126088 | 186910270 | general transcription factor IIH, polypeptide 5 |
| Atxn7l3 | 3.11 | 3.88 | XM_213493.3 | 392351635 | ataxin 7-like 3, transcript variant 2 |
| MAP1B | 3.166 | 2.525 | NM_019217 | 158749619 | microtubule-associated protein 1B |
| Sypl1 | 3.106 | 1.868 | NM_001014263 | 62079260 | synaptophysin-like 1 |
| RPL18A | 3.09 | 5.68 | NM_212510.1 | 47059005 | Rattus norvegicus ribosomal protein L18A (Rpl18a) |
| RGD1565784 | 3.05 | 2.47 | XM_001059479.1 | 157817212 | PREDICTED: Rattus norvegicus RGD1565784 (predicted) (RGD1565784_predicted) |
| TMEFF1 | 3.03 | 5.89 | NM_023020.2 | 121583853 | Rattus norvegicus transmembrane protein with two follistatin-like domains 1 (Tmeff1) |
| Fabp7 | 3 | 3.62 | NM_030832.1 | 402744885 | fatty acid binding protein 7, brain |
| Map1lc3a | 3 | 1.68 | NM_199500 | 377652334 | microtubule-associated protein 1 light chain 3 alpha |
| LOC499793 | 2.97 | 5.51 | XM_575129.1 | 62645054 | similar to 60S ribosomal protein L12 |
| Cbr1 | 2.94 | 2.98 | NM_019170.2 | 46048534 | carbonyl reductase 1 (Cbr1) |
| PEA15A | 2.896 | 2.177 | NM_001013231.1 | 61557369 | phosphopro cytoch |
| SLC25A11 | 2.89 | 4.96 | NM_022398.1 | 11693169 | solute carrier family 25 (mitochondrial carrier; oxoglutarate carrier) |
| Aplp1 | 2.89 | -2.08 | XM_001076030.1 | 198041598 | similar to Amyloid beta (A4) precursor-like protein 1 (predicted) |
| Cox7a2l | 2.88 | 5.51 | NM_001106704 | 157821820 | cytochrome c oxidase subunit VIIa polypeptide 2 like |
| BAX | 2.87 | 3.103 | NM_017059 | 386869282 | Bcl2-associated X protein |
| S100a1 | 2.85 | 2.51 | NM_001007636.1 | 218749895 | S100 calcium binding protein A1 (S100a1) |
| Rpl7 | 2.82 | 2.53 | NM_001100534 | 198278504 | ribosomal protein L7 |
| RGD1564372 | 2.77 | 4.31 | XM_344301.1 | 34869098 | similar to RIKEN cDNA 1110003E01 (predicted) (RGD1564372_predicted) |
| Dpp9 | 2.77 | 2.28 | XM_001061312.1 | 392342223 | dipeptidyl peptidase 9 |
| IL-17D | 2.77 | 2.54 | XM_577439.1 | 62661954 | similar to IL-17D (LOC502011) |
| RGD1565358 | 2.76 | 2.29 | XM_235062.2 | 109480494 | genes associated with retinoid-IFN-induced mortality 19 (predicted) |
| C1QL1 | 2.76 | 2.8 | NM_001108838 | 157819268 | complement component 1,q subcomponent-like 1 |
| Lrrc73 | 2.75 | 5.11 | NM_001024360.1 | 119310198 | eucine rich repeat containing 73 |
| Actb | 2.75 | 6.06 | NM_031144.2 | 42475962 | beta actin |
| Mapre1 | 2.742 | 1.579 | NM_138509.3 | 78097099 | microtubule-associate |
| RAB3A | 2.72 | 3.33 | NM_013018.2 | 61098194 | RAB3A, member RAS oncogene family |
| LOC301444 | 2.72 | 3.83 | NM_001047872 | 114145717 | pseudogene for diazepam binding inhibitor 1 (LOC301444) |
| LYPLA2 | 2.71 | 4.7 | NM_031342.1 | 13786177 | lysophospholipase 2 (Lypla2) |
| NDUFB8 | 2.66 | 5.9 | NM_001106360 | 187937126 | NADH dehydrogenase (ubiquinone) 1 beta subcomplex 8 |
| Hm13 | 2.65 | 3.74 | NM_001107789 | 157823208 | histocompatibility 13 |
| Hdac3 | 2.623 | 2.343 | NM_053448.1 | 16758191 | histone deacetylase 3 |

| | | | | | |
|---|---|---|---|---|---|
| SNAP91 | 2.61 | 4.12 | NM_031728.1 | 13994176 | synaptosomal-associated protein 91 (Snap91) |
| TERF2 | 2.61 | 1.54 | NM_001242355.1 | 334285045 | telomeric repeat binding factor 2, transcript variant 2 |
| Nras | 2.6 | 4.8 | NM_080766.1 | 18158430 | neuroblastoma ras oncogene (Nras) |
| LOC691672 | 2.6 | 4.06 | XM_001079224.1 | 109513656 | similar to Discs large homolog 5 (Placenta and prostate DLG) |
| MLLT3 | 2.604 | 3.286 | NM_053718.1 | 192447427 | myeloid/lymphoid or mixed lineage-leukemia translocation to 3 homolog (Drosophila) |
| Zfp513 | 2.59 | 4.67 | NM_001012110.1 | 58865789 | zinc finger protein 513 (Zfp513) |
| Mgrn1 | 2.57 | 4.76 | NM_001013964.1 | 62078656 | mahogunin ring finger 1, E3 ubiquitin protein ligase |
| Ndufb2 | 2.56 | 2.54 | XM_001070173.1 | 219277691 | NADH dehydrogenase (ubiquinone) 1 beta subcomplex |
| Stk11 | 2.55 | 4.6 | XM_234901.4 | 157823728 | downstream of Stk11 (predicted) (Dos_predicted) |
| Hes5 | 2.55 | 4.53 | NM_024383.1 | 13242286 | hairy and enhancer of split 5 (Drosophila) (Hes5) |
| VAMP8 | 2.491 | 4.002 | NM_031827 | 13929181 | vesicle-associated membrane protein 8 |
| Lmo3 | 2.43 | 3.91 | NM_139112.1 | 20806164 | LIM domain only 3 (Lmo3) |
| Mkrn3 | 2.41 | 3.8 | XM_001057262.1 | 392337728 | makorin, ring finger protein, 3 |
| Gnal | 2.357 | 2.641 | NM_001191836 | 300797436 | guanine nucleotide binding protein, alpha, stimulating, olfactory type |
| NDUFB3 | 2.259 | 3.999 | NM_001106912 | 157824070 | NADH dehydrogenase (ubiquinone) 1 beta subcomplex 3 |
| Lemd2 | 2.18 | 4.78 | NM_001039032.1 | 84781657 | Rattus norvegicus LEM domain containing 2 (Lemd2) |
| Ube2c | 2.18 | 1.86 | NM_001106542 | 157818348 | ubiquitin-conjugating enzyme E2C |
| Efna2 | 2.15 | 2.53 | NM_001168670 | 274327666 | ephrin A2 (Efna2) |
| LOC361128 | 2.08 | 4.17 | XM_341415.2 | 62662451 | similar to TR4 orphan receptor associated protein TRA16 (LOC361128) |
| Hnrpa2b1 | 1.97 | 4.15 | XM_342684.3 | 157265558 | heterogeneous nuclear ribonucleoprotein A2/B1 (predicted) (Hnrpa2b1_predicted) |
| MTAP2 | 1.939 | 1.862 | NM_013066.1 | 6981181 | microtubule-associated protein 2 |
| Sdhb | 1.82 | 2.12 | NM_001100539 | 209915613 | succinate dehydrogenase complex, subunit B, iron sulfur (Ip) |

Supplemental Table 2: **TRF2-S targets related to axonogenesis**

mRNA identified in DIV 13 cortical axons[8]

| | |
|---|---|
| RPL18A | Ribosomal protein L18A |
| RPL13A | Ribosomal protein L13A |
| LOC499793 | 60S ribosomal protein L12 |
| Rpl7 | Ribosomal protein L7 |
| Hnrpa2b1 | Heterogeneous nuclear ribonucleoprotein A2/B1 |
| NNAT | Neuronatin |
| MAP1B | Microtubule-associated protein 1B |
| Map1lc3a | Microtubule-associated protein 1 light chain 3 alpha |
| ATP5G3 | ATP synthase, H+ transporting, mitochondrial Fo complex, subunit 9 |
| Cox7b | Cytochrome c oxidase subunit VIIb |
| COX7A2L | Cytochrome c oxidase subunit VIIa polypeptide 2 |
| CAMK2N2 | Calcium/calmodulin-dependent protein kinase II inhibitor 2 |
| GNG10 | Guanine nucleotide binding protein (G protein) |
| TXN1 | Thioredoxin 1 |
| NME2 | Non-metastatic cells 2 |
| NREP | Neuronal regeneration related protein |
| LOC691672 | Discs large homolog 5 |

mRNA indentified in embryonic axons[11]

| | |
|---|---|
| RPL18A | Ribosomal protein L18A |
| RPL13A | Ribosomal protein L13A |
| LOC499793 | 60S ribosomal protein L12 |
| Rpl7 | Ribosomal protein L7 |
| NNAT | Neuronatin |
| MAP1B | Microtubule-associated protein 1B |
| Map1lc3a | Microtubule-associated protein 1 light chain 3 alpha |
| MAPRE1 | Microtubule-associated protein |
| MTAP2 | Microtubule-associated protein 2 |
| Nckap1 | NCK-associated protein |
| ATP5G3 | ATP synthase, H+ transporting, mitochondrial Fo complex, subunit 9 |
| SLC25A11 | Solute carrier family 25 (mitochondrial carrier; oxoglutarate carrier) |
| COX7A2L | Cytochrome c oxidase subunit VIIa polypeptide 2 |
| BAX | Bcl2-associated X protein |
| Ndufb2 | NADH dehydrogenase (ubiquinone) 1 beta subcomplex |
| NDUFB3 | NADH dehydrogenase (ubiquinone) 1 beta subcomplex 3 |
| Cox7b | Cytochrome c oxidase subunit VIIb |
| TOMM22 | Outer mitochondrial membrane 22 homolog (yeast) |
| RAB3A | RAB3A |
| APLP1 | Amyloid beta (A4) precursor-like protein 1 |
| RAB2 | RAB2 |
| SYPL | Synaptophysin-like 1 |
| GDI1 | Guanosine diphosphate dissociation inhibitor 1 |
| GNG10 | Guanine nucleotide binding protein (G protein) |
| NREP | Neuronal regeneration related protein |
| Akt1 | Thymoma viral proto-oncogene 1 |
| Brsk1 | BR serine/threonine kinase 1 |

| | |
|---|---|
| PPP2R5B | Protein phosphatase 2 |
| YY1 | YY1-associated factor 2 |
| HMGB2 | High mobility group box 2 |
| TXN1 | Thioredoxin 1 |
| CIC | Capicua homolog (Drosophila) |

Supplemental Table 3

## Oligomer pairs for biotin pull-down assays

| | Forward | Reverse |
|---|---|---|
| **Trf2s:** | | |
| 5'UTR | CCAAGCTTCTAATACGACTCACTATAGGGAGAAACAGCGGGCCCTGCTTC | CGTGTTCGACCGCCGAG |
| CR1 | CCAAGCTTCTAATACGACTCACTATAGGGAGAATGGCGGGAGGAGGCG | ATTTTTGAAGCCTTTTCAAATTCTT |
| CR2 | CCAAGCTTCTAATACGACTCACTATAGGGAGATTTGTATCAAAAACAAAGAATTTGA | TTAGGGCTGTTCCACTTG |
| 3'UTR | CCAAGCTTCTAATACGACTCACTATAGGGAGACGGGCTTGAAGAAAAGGAAG | CCCGCACTTCAAACAGCT |
| | | |
| Clip-WT | CCAAGCTTCTAATACGACTCACTATAGGGAGAGGTCTGCGGGCCTTTCGGAGTAGCCGGTACCGGGACTTCAGGCAGATCCG | CGGATCTGCCTGAAGTCCCGGTACC |
| Clip-MT1 | CCAAGCTTCTAATACGACTCACTATAGGGAGAGGT CTGCGGGCCTAAGCCTCATCCCGGTACCGGGACTTCAGGCAGATCCG | CGGATCTGCCTGAAGTCCCGGTACC |
| Clip-MT2 | CCAAGCTTCTAATACGACTCACTATAGGGAGAGGT CTGCGGGCCTTTCGGAGTAGCCGGTACCGGGACTTCACCGTCTAGCG | CGCTAGACGGTGAAGTCCCGGTACC |
| ClipMT1+2 | CCAAGCTTCTAATACGACTCACTATAGGGAGAGGT CTGCGGGCCTAAGCCTCATCCCGGTACCGGGACTTCACCGTCTAGCG | CGCTAGACGGTGAAGTCCCGGTACC |
| | | |
| **Aplp1** | | |
| 5'CR1 | CCAAGCTTCTAATACGACTCACTATAGGGAGAGCAGGGCCGGGCGGGAGCGCA | AGGCAATGGAAGGGCACAACC |
| CR2 | CCAAGCTTCTAATACGACTCACTATAGGGAGAGGTTGTGCCCTTCCATTGCCT | GTGGGAGTGACTCTGCTGACC |
| CR3 | CCAAGCTTCTAATACGACTCACTATAGGGAGAGGTCAGCAGAGTCACTCCCAC | CTCAGGGCCATCAGAACTCTC |
| CR4 | CCAAGCTTCTAATACGACTCACTATAGGGAGAGAGAGTTCTGATGGCCCTGAG | GGAAGGGTCACTGGGGCATCT |
| CR5 | CCAAGCTTCTAATACGACTCACTATAGGGAGAAGATGCCCCAGTGACCCTTCC | AGCTAGGGTTGGGGTCAAGGT |
| 3'UTR | CCAAGCTTCTAATACGACTCACTATAGGGAGAGAAGAACGACCTTGACCCCAACCCT | GAAGAGGACTTTATTGGGATGTTAG |
| | | |
| **Rab3a** | | |
| 5'CR1 | CCAAGCTTCTAATACGACTCACTATAGGGAGAGAGCCTCCACCGCCGCCTGCA | ACATTAGAATGAAGCCCATGG |
| CR2 | CCAAGCTTCTAATACGACTCACTATAGGGAGACCATGGGCTTCATTCTAATGT | GGTGGGGAAGGGAAGTGTGGT |
| 3'UTR | CCAAGCTTCTAATACGACTCACTATAGGGAGAACCACACTTCCCTTCCCCACC | TTCTCTCTTTAATGTCAAGGACTGC |
| | | |
| **App** | | |
| 887-1150 | CCAAGCTTCTAATACGACTCACTATAGGGAGAtgcagatgggagtgaagacaaag | tggaaatgggcatgttcattctc |
| 1944-2150 | CCAAGCTTCTAATACGACTCACTATAGGGAGAccgccctgctgccgaccgagga | cttcttcagcatcaccaaggtg |
